# Immunotherapeutic Potential of T Memory Stem Cells

**DOI:** 10.3389/fonc.2021.723888

**Published:** 2021-09-17

**Authors:** Yujie Li, Dengqiang Wu, Xuejia Yang, Sufang Zhou

**Affiliations:** ^1^Department of Biochemistry and Molecular Biology, School of Pre-Clinical Science, Guangxi Medical University, Nanning, China; ^2^National Center for International Research of Bio-targeting Theranostics, Guangxi Key Laboratory of Bio-targeting Theranostics, Collaborative Innovation Center for Targeting Tumor Diagnosis and Therapy, Guangxi Medical University, Nanning, China

**Keywords:** T memory stem cells, stemness, tumor immunotherapy, HIV, autoimmune diseases

## Abstract

Memory T cells include T memory stem cells (T_SCM_) and central memory T cells (T_CM_). Compared with effector memory T cells (T_EM_) and effector T cells (T_EFF_), they have better durability and anti-tumor immunity. Recent studies have shown that although T_SCM_ has excellent self-renewal ability and versatility, if it is often exposed to antigens and inflammatory signals, T_SCM_ will behave as a variety of inhibitory receptors such as PD-1, TIM-3 and LAG-3 expression, and metabolic changes from oxidative phosphorylation to glycolysis. These changes can lead to the exhaustion of T cells. Cumulative evidence in animal experiments shows that it is the least differentiated cell in the memory T lymphocyte system and is a central participant in many physiological and pathological processes in humans. It has a good clinical application prospect, so it is more and more important to study the factors affecting the formation of T_SCM_. This article summarizes and prospects the phenotypic and functional characteristics of T_SCM_, the regulation mechanism of formation, and its application in treatment of clinical diseases.

## Introduction

Immunotherapy has become one of the most promising strategies in cancer treatment, and has shown good efficacy in clinical trials (1). In particular, chimeric antigen receptor-engineered T cells (CAR-T) can specifically and effectively kill tumor cells, bringing new hopes for the treatment of patients with malignant tumors (2–7). However, whether it is traditional immune cell therapy or new CAR-T cells and T-cell receptor T cells (TCR-T) therapy, all are based on terminally differentiated effector T (T_TE_) cells, making it difficult to exert long-lasting anti-tumor effects in the body (8). Adoptive T cell therapy (ACT) is the *in vitro* expansion and reinfusion of tumor-reactive T cells, and is a potential treatment method for the treatment of advanced cancer (9–14). In infections and cancers, T lymphocytes expand and differentiate into effector cells and memory cells that clear pathogens. These cells can survive for a long time and ensure that they have a protective effect against pathogens when they are re-attacked by antigens (15). Human T lymphocytes are generally divided into naive T cell (T_N_), central memory T cell (T_CM_), effector memory T cell (T_EM_) and effector T cell (T_EFF_). In 2005, in the study of graft *versus* host disease (GVHD) in mice, a group of special memory T cell subsets with super proliferation and differentiation ability was observed for the first time. It produces persistent graft-*versus*-host disease, which the researchers named “stem like memory T cells” (T_SCM_) (16). Studies have shown that adoptively infused young T cells can self-renew and differentiate in mice, having the ability to survive for a long time, and exhibit significantly better anti-tumor capabilities than T_TE_ cells. The progressive differentiation of T lymphocytes leads to a gradual loss of function and therapeutic potential. These studies suggest that poorly differentiated immune cells may have more application potential in clinical treatment (17–21).

**Figure 1:**
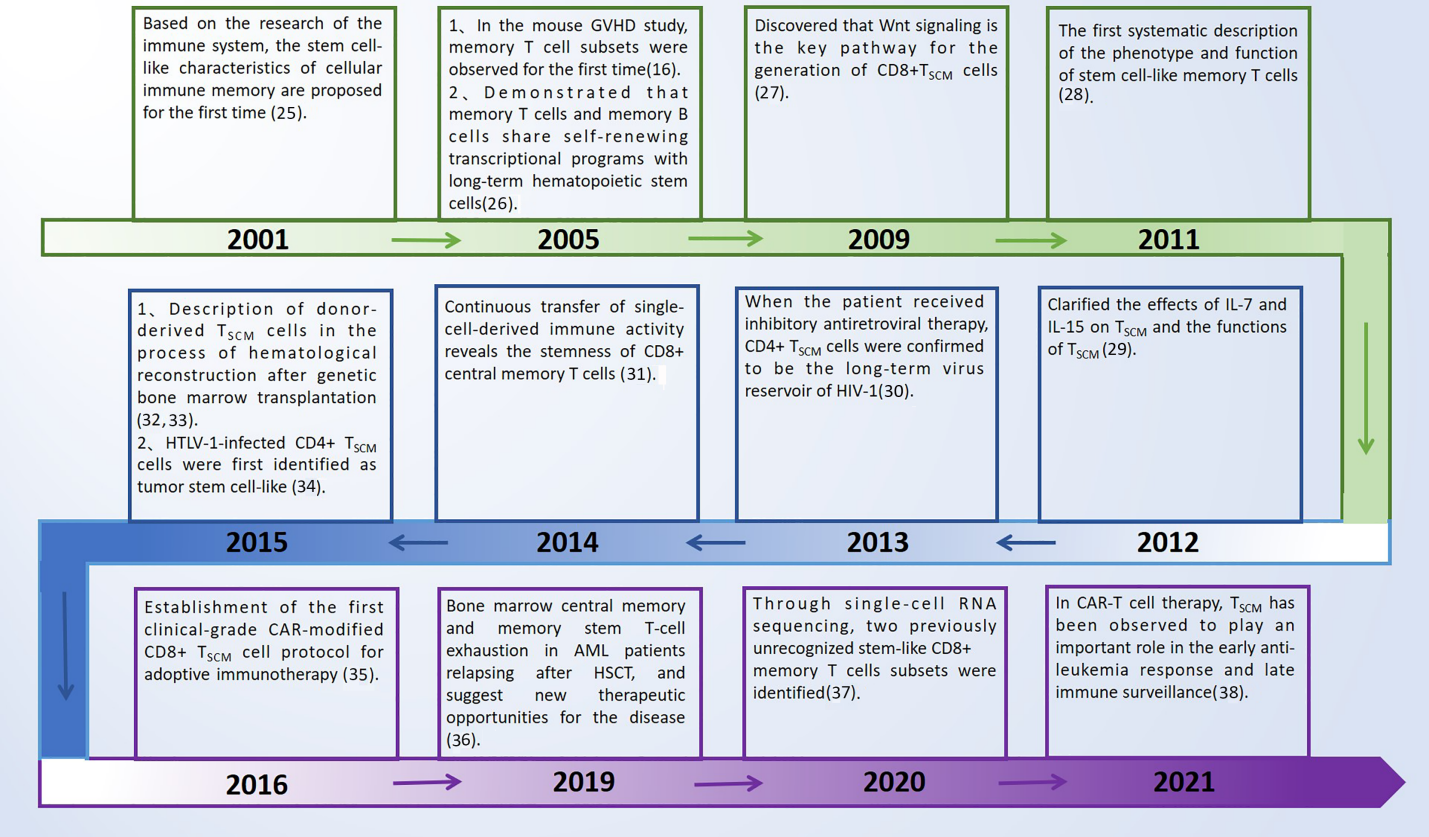
Key discoveries on T_SCM_ cells. GVHD, graft *versus* host disease; T_SCM_ cells, T memory stem cells; HTLV-1, human T cell lymphotropic virus type 1; HIV-1, human immunodeficiency virus type 1; CAR-T, chimeric antigen receptor-engineered T cells; AML, acute myeloid leukemia; HSCT, hematopoietic stem cell transplantation.

T_SCM_ cells have great potential in overcoming the limitations of current T cell-based immunotherapy (22–24). In mouse tumor models and human hematopoietic stem cell transplantation (HSCT) patients, T_SCM_ cells have higher antitumor activity and survival rate. However, the proportion of T_SCM_ cells in peripheral blood is low, which limits its application in immunotherapy. In this review, we summarize the latest findings, and discuss in depth the phenotype, function, differentiation mechanism and clinical application of memory T cells. It is hoped that using the therapeutic potential of T_SCM_ cells for adoptive immunotherapy provides new ideas. The conceptual work and key discoveries that formed this field of investigation are shown in **Figure 1** (25–38), which mainly summarizes the main discoveries in the process of T_SCM_ cell research in recent years and the new research on the occurrence and development of diseases, some of which are introduced in articles.

## Phenotypic and Functional Characteristics of Memory T Cells

T_SCM_ is a T cell subset with self-renewal ability and pluripotency potential. This group of memory T cells can play the role of acquired immune function in the process of the body’s fight against viruses or tumors (36, 39). T cell populations are classified by some surface markers, and distinguished according to their functions and sources, and the production of their effector cytokines. Memory T cells can be divided into T_CM_ and T_EM_. T_EM_ cells and T_CM_ cells circulate in the blood and target the secondary lymphoid tissues. The degree of differentiation of T_CM_ cells is lower than that of T_EM_ and effector cells, and its telomeres are found to be longer, and the expression of perforin, granzyme and other effector molecules is lower (40). In addition, the T_SCM_ pool should be limited to lymph nodes and secondary lymphoid organs, which are T cells that have antigen experience. The current research results also show that T_CM_ has the function of T memory stem cells. T_CM_ has stronger immune replacement ability and stronger survival ability *in vivo* than T_EM_ cells. T_SCM_ is developed from naive T cells in a resting state. It is a group of cells between T_N_ and T_CM_. It also has the characteristics of T_N_ cells and memory T cells (T_M_), and then differentiates into T_CM_ and T_EM_. Good et al. (41) used single-cell mass cytometry to track the proliferation history of T cells. By analyzing the changes in phenotype and protein expression of T cells at different times and in different division states, it assisted in confirming the T cell differentiation theory: T_N_ → T_SCM_ → T_CM_ → T_EM_. It is worth noting that only naive T cells and T_SCM_ cells can reconstruct the heterogeneity of the entire memory T cell subset. At present, malignant tumors are one of the important diseases threatening human health, and there is no effective method to treat them. Due to their own characteristics, T_SCM_ cells have shown their strong potential for tumor therapy.

According to the different expressions of cell surface chemokine receptor (CCR7) and lymph node homing molecules (CD62L), memory T cells are divided into T_CM_ and T_EM_. T_CM_ highly expresses CCR7 and CD62L, homing to secondary lymphoid organs, but low expression in T_EM_, which preferentially transports to peripheral tissues and mediates rapid effector functions. T_SCM_ cells express naive cell phenotypes (CD45RA, CD62L, CCR7, CD95, CD27, CD122), and are characterized by rapid response to antigens, expression of a variety of effector molecules, and generation of memory effector cells. CD45RA^+^ is closely related to its memory ability. Naive cells express two molecules CD27 and CD45RA at the same time. Memory and effector cells only express CD27 or CD45RA respectively. T_SCM_ cells highly express IL-2, IFN-γ, TNF-α, Bcl- 2. IL-7 and other molecules related to early differentiation of T cells, low expression of CD57 and other molecules related to T cell senescence, showing stronger degranulation ability and the ability to produce inflammatory cytokines. Recent studies have found that by detecting the expression of CD122 or CXCR3 in healthy people by flow cytometry, the T_SCM_ CD122^hi^-expressing subset demonstrate greater proliferation, greater multipotency and enhanced polyfunctionality with higher frequencies of triple positive (TNF-α, IL-2, IFN-γ) cytokine-producing cells upon exposure to recall antigen. The cell proliferation and multifunctional cytokine production of the T_SCM_ CXCR3^lo^ population are also significantly increased (42). Loss of CXCR3 promotes stem-like memory precursor differentiation (43). According to these surface markers, T_SCM_ cells can be accurately distinguished. T_SCM_ cells represent a subset of minimally differentiated T cells, which are characterized by phenotypic and functional characteristics that connect naive and conventional memory cells.

The above mainly describes the surface markers of human T cell subsets. In addition to the specific T cell receptor (TCR), both human and murine T_SCM_ express common markers of memory T cells (mouse CD62L, human CCR7, human CD45RO), and anti-apoptotic marker molecules (Bcl- 2), the cytokine receptor markers related to survival and proliferation CD122 (co-receptor of IL-2, IL-7 and IL-15) and CD127 (IL-7 receptor), stem cell marker (Sca-1). Human and murine T cell subsets are defined by different phenotypes (16, 27) (**Table 1**).

**Table 1:** Phenotypic markers of memory T cells.

Subset	Phenotype (Human)	Phenotype (Mice)	Characteristics
**T_N_**	CD45RA^+^,CD45RO^-^,CCR7^+^,CD62L^+^,CD127^+^,CD122^+^,CD27^+^,CD44^-^,CD28^+^,CD43^-^,CD95^-^,CD57^-^,CD58^-^,CD11α^-^,(IL-7Rα)^+^,CXCR3^-^,(IL-2Rβ)^-^	CD44^-^, CD62L^+^, CCR7^+^, CXCR5^-^, CXCR3^-^	Multidirectional differentiation ability
**T_SCM_**	CD45RA^+^,CD45RO^-^,CCR7^+^,CD62L^+^,CD127^+^,CD122^+^,CD27^+^,CD44^+/-^,CD28^+^, CD43^-^,CD95^+^,CD57^-^,CD58^+^,CD11α^+^,(IL-7Rα)^+^,CXCR3^+^,(IL-2Rβ)^+^	CD44^-^, CD62L^-^,(Sca-1)^+^,CD122^+^,(Bcl-2)^+^,CCR5^+^,CXCR3^+^	Self-renewal capacity and multipotency
**T_CM_**	CD45RA^-^,CD45RO^+^,CCR7^+^,CD62L^+^,CD127^+^,CD122^+^,CD27^+^,CD44^+^,CD28^+^, CD43^-^,CD95^+^,CD57^-^,CD58^+^,CD11α^+^,(IL-7Rα)^+^,CXCR3^+^,(IL-2Rβ)^+^	CD44^+^, CD62L^+^, CCR7^+^	Long-lasting immune memory
**T_EM_**	CD45RA^-^,CD45RO^+^,CCR7^-^,CD62L^-^,CD127^+^,CD122^+^,CD27^+/-^,CD44^+^,CD28^+/-^,CD43^+/-^,CD95^+^,CD57^+/-^,CD58^+^,CD11α^+^,(IL-7Rα)^+/-^, CXCR3^+^,(IL-2Rβ)^+^	CD44^+^, CD62L^-^, CCR7^-^	Immediate effector function
**T_TE_**	CD45RA^-^,CD45RO^-^,CCR7^-^,CD62L^-^,CD127^+^,CD122^-^,CD27^-^,CD44^-^,CD28^-^,CD43^+^,CD95^+^,CD57^+^,CD58^+^,CD11α^+^,(IL-7Rα)^-^,CXCR3^-^,(IL-2Rβ)^+^	CD44^-^, CD62L^+^	Terminally differentiated effector T cells

“+” positive expression; “−” negative expression; T_N_, naive T cell; T_SCM_, stem cell memory T cell; T_CM_, central memory T cell; T_EM_, effector memory T cell; T_TE_, terminal effector T cell.

## Development of T_SCM_ Cells

### Manipulation to Produce T_SCM_ Cells in *(Ex) Vivo*

**Figure 2:**
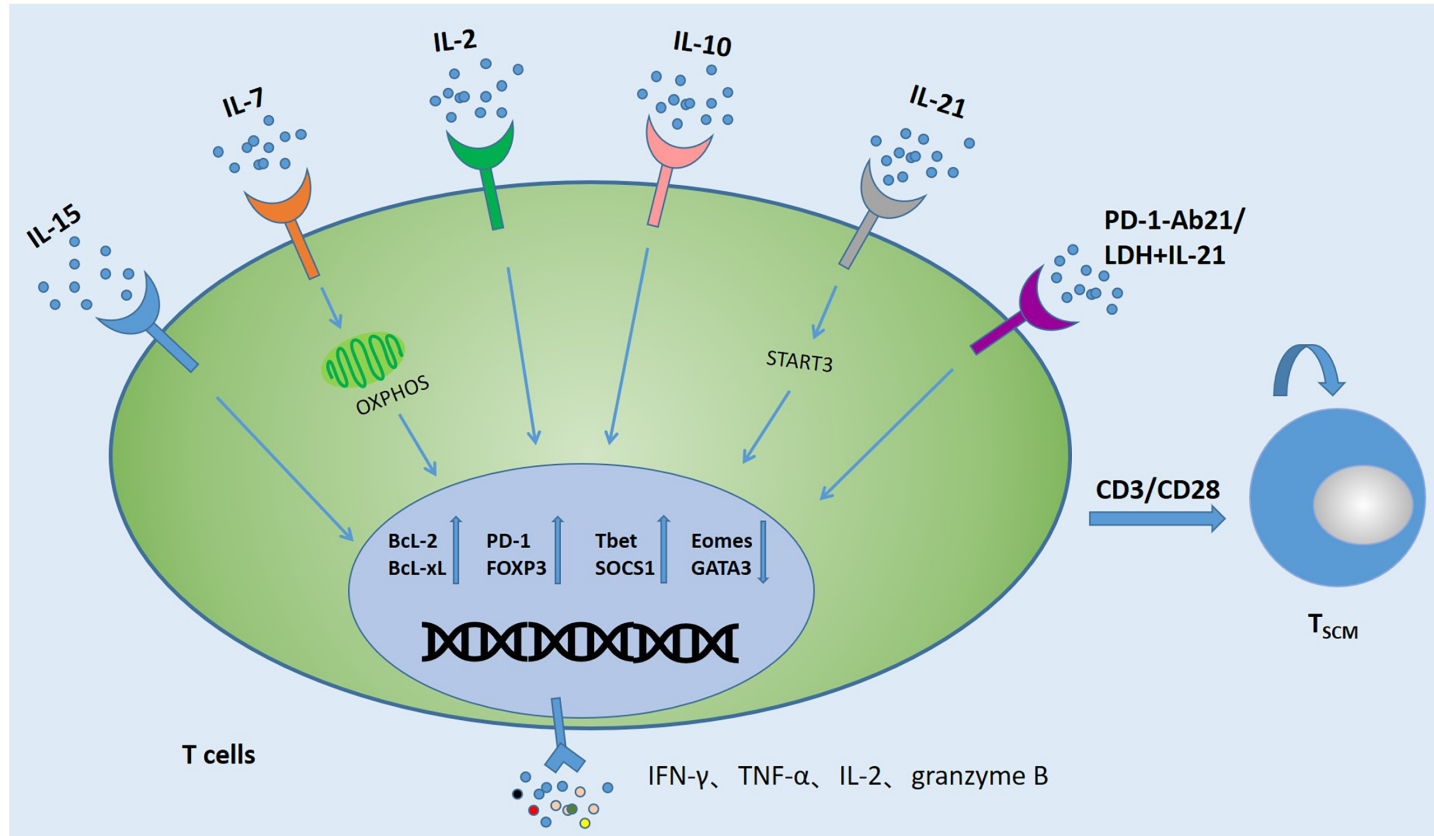
Several strategies to induce the generation of T_SCM_. Activating T cells (CAR-T cells, TCR-T cells, TILs, VSTs) with anti-CD3/CD28 antibodies and co-cultivating them with cytokines or combined with PD-1 and LDH can promote the production of T_SCM_ cells and change the expression levels of related anti-apoptotic proteins and metabolic molecules. In addition, the expression of TNF-α, IFN-γ, IL-2 and Granzyme B also increased significantly.

The relative scarcity of circulating T_SCM_ cells limits their use in tumor therapy, which has led to manufacturing protocols that expand this cell type *in vitro*. As an important participant in the function of T cells, cytokines play an important role in the maintenance and expansion of T_SCM_ subset. Recently reported related cytokines that can promote T_SCM_ expansion are shown in **Figure 2**. A large number of studies have shown that adding different cytokines to the immune cell culture system can make it differentiate into memory or effector T cells (44–47). γc-cytokine IL-2, as a T cell growth factor, is still the most common cytokine used to expand therapeutic T cell products for patients (29, 48–50). γc-cytokine IL-2, as a T cell growth factor, is still the most common cytokine used to expand therapeutic T cell products for patients. However, high IL-2 levels reduced the overall production of early memory T cells by reducing central memory T cells and augmenting effectors. The number of early memory T cells in the T cell subset could be increased by simply reducing the amount of IL-2 (51). In the *in vitro* expansion process, repeated use of IL-2 to stimulate T cells would also cause T cell depletion and reduced T cell persistence (52). IL-7 could also promote the proliferation of T_SCM_ cells by down-regulating the expression of programmed cell death protein 1 (PD-1) and Foxp3, and promoted the ability of CD4^+^ T cells to produce IFN-γ, IL-2, TNF-α and granzyme B. The involvement of STAT5 in IL-7-induced polyfunctionality, this the polyfunctional phenotype driven by IL-7 is associated with increased histone acetylation effector gene promoters and reveals previously unknown characteristics of IL-7 (53–56). The current study, CAR-T cells expanded in IL-15 (CAR-T/IL-15) preserved a less-differentiated T_SCM_ phenotype, defined by expression of CD62L^+^CD45RA^+^CCR7^+^, as compared to cells cultured in IL-2 (CAR-T/IL-2). What’s more, CAR-T/IL-15 cells exhibited reduced expression of exhaustion markers, higher anti-apoptotic properties, and increased proliferative capacity when it was attacked by antigens (57). The combined use of IL-7 and IL-15 can preserve the T_SCM_ phenotype and enhance the effectiveness of CAR-T cells (11, 29, 58–61). IL-21 was critical for the long-term maintenance and functionality of CD8^+^T cells and the control of chronic lymphocytic choriomeningitis virus (LCWV) infection in mice. In the process of chronic infection, cell-autonomous IL-21 receptor (IL-21R)–dependent signaling by CD8^+^ T cells was required for sustained cell proliferation and cytokine production (62, 63). IL-21 also can promote the generation of T_SCM_ cells. It activates the Janus kinase signal transducer and activator of transcription 3 pathway by upregulating signal transducer and activator of transcription 3 phosphorylation and thereby promoting the expression of T-bet and suppressor of cytokine signaling 1, while decreasing the expression of eomesodermin (Eomes) and GATA binding protein 3 (64). In the absence of IL-10, IL-21 or STAT3, virus-specific CD8^+^ T cells (VSTs) maintain the terminal effect (T_E_) differentiation state and couldn’t mature into self-renewing T_CM_ cells. The maturation of protective memory T cells and memory CD8^+^ T cell precursors was an active process that depended on the IL-10-IL-21-STAT3 signal (64, 65). Whether the formation of T_SCM_ depends on this pathway still needs further research, but it provides new ideas for subsequent research. Lactate dehydrogenase (LDH) inhibition combined with IL-21 could increase the formation of T_SCM_ cells, thereby producing more profound antitumor responses and prolonging the survival time of the host (66). In addition, a new study found that by fusion of IL-21 to anti-PD-1 antibody, IL-21 can target tumor-reactive T cells to promote T_SCM_ production. PD-1Ab21 therapy has shown greater antitumor effects in established tumor-bearing mice (67). At present, a large number of experiments have confirmed that these cytokines can promote the production of T_SCM_ and have potential antitumor effects. However, the mechanism of using cytokines, drugs and checkpoint blockade to promote the differentiation of memory T cells remains to be studied.

### Oxidative Metabolic Pathway of T_SCM_ Cells

**Figure 3:**
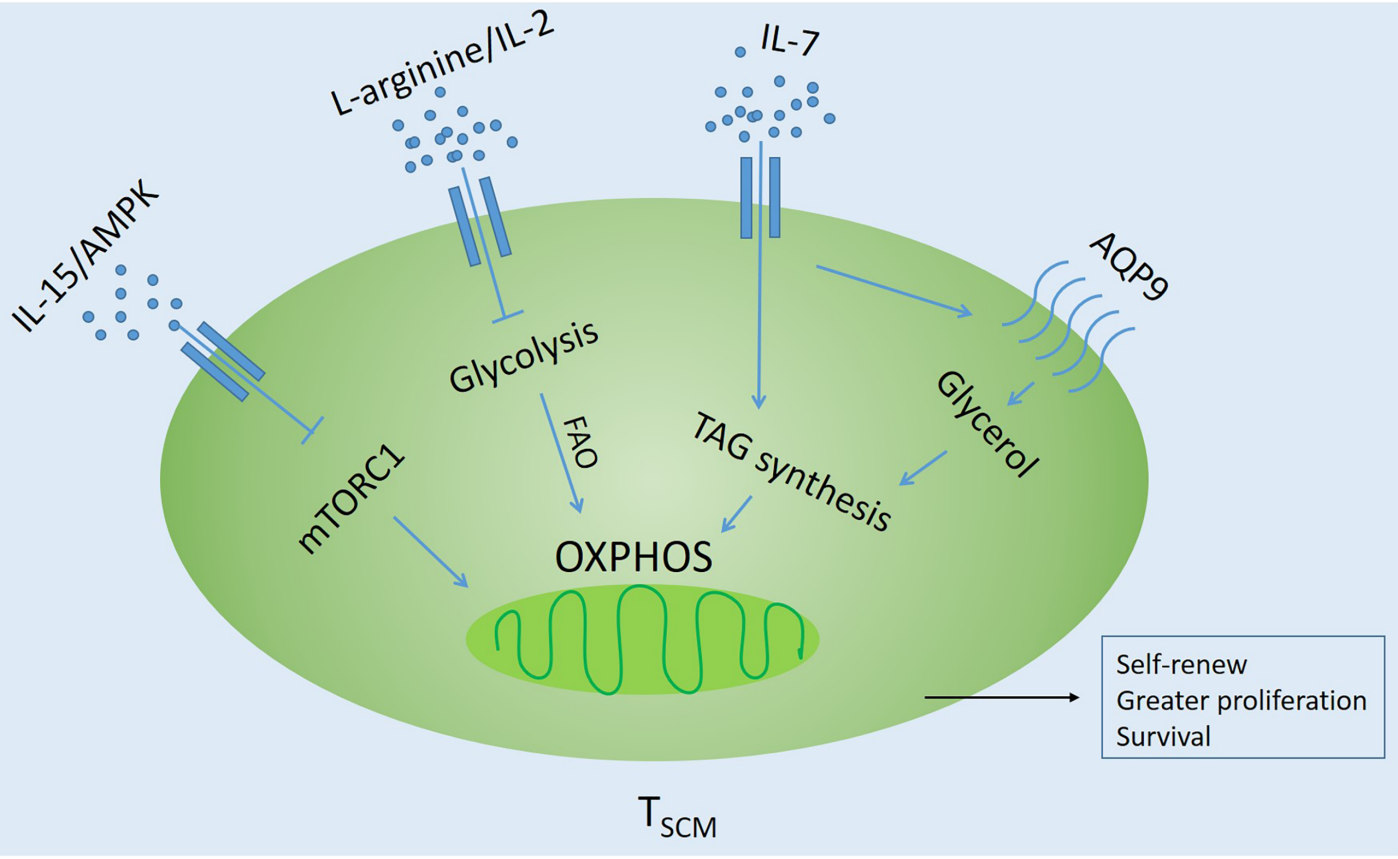
Influencing factors regulating oxidative metabolism of T_SCM_ Cells. Inhibit glycolysis through different pathways and promote fatty acid oxidation (FAO)-dependent oxidative phosphorylation (OXPHOS). AQP9, Glycerol channel aquaporin 9; AMPK, AMP-activated protein kinase; TAG, triglyceride; mTORC1, Rapamycin Complex 1.

The naive T cells in the circulation are quiescent and have low metabolic requirements. They mainly use oxidative phosphorylation (OXPHOS) to produce ATP (53, 68). Generally speaking, differentiated T cells use glycolysis to proliferate, while memory T cells tend to use fatty acid oxidation (FAO)-dependent oxidative phosphorylation (OXPHOS) to produce ATP, which helps to perform long-lasting antitumor response in the tumor microenvironment (69–74). In the tumor microenvironment, tumor cells inhibit the metabolic reprogramming of T cells by competitively using glycolysis, so that the formation of memory T cells is inhibited (75, 76). It is reported that important transcription factors and cytokines, as well as MEKi and other inhibitors in the process of T cell differentiation, induce the generation of T_SCM_ by regulating T cell-related metabolic enzymes (77–79) (**Figure 3**).

Signals from TCR, costimulatory molecules, and growth factors lead to the activation of signaling pathways that promote transcriptional programs that are critical to effector function (80–82). In memory T cells, cellular stress, such as growth factor deprivation or a low ratio of ATP/AMP, will activate AMP-activated protein kinase (AMPK) and inhibit mTOR signaling (83). IL-15 also showed a similar function (57, 83).

Good et al. (41, 84) have proved through a large number of experiments that blocking the mTOR pathway by adding inhibitors can allow T cells to differentiate towards T_SCM_-like cells, such as ITK (IL-2-inducible T-cell kinase), TWS119 and BTK (Bruton’s tyrosine kinase) inhibitors. In addition, the glycolytic function of T_SCM_ cells is reduced, and different inhibitors promote the *in vitro* generation of T_SCM_-like cells with unique metabolic characteristics and retained polyfunctionality. It is worth noting that the drug-induced T_SCM_ cells have superior functional characteristics and self-renewing capacity after adoptive transfer. The research compound Akt inhibitor VIII inhibits AKT *in vitro*, which can preserve the differentiation and function of minor histocompatibility antigen (MIHA)-specific CD8^+^ T cells. Moreover, transcriptome profiling revealed that AKT-inhibited CD8^+^T cells clustered closely to naturally occurring stem cell-memory CD8^+^ T cells. Moreover, AKT-inhibited MiHA-specific CD8^+^ T cells showed increased polyfunctionality with co-secretion of IFN-γ and IL-2 upon antigen recall (79). Glycerol channel aquaporin 9 (AQP9) deficiency could impair the entry of glycerol into memory CD8^+^ T cells for fatty acid esterification and triglyceride (TAG) synthesis and storage. While IL-7 could induce expression of the AQP9 in virus-specific memory CD8^+^ T cells, but not naive cells. AQP9 is essential for their long-term survival. TAG synthase could restore the survival of lipid storage and memory T cells through ectopic expression, and it was found that TAG synthase is the central component of IL-7-mediated survival of human and mouse memory CD8^+^ T cells (75). Three transcription factors, BAZ1B, PSIP1 and TSN, could regulate the level of L-arginine and promoted the survival of T cells. Activated T cells transform from glycolysis to oxidative phosphorylation, which promotes the production of T_SCM_ with higher survival ability and has antitumor activity in mouse models (85). Recent new studies have found that T_SCM_ induced by Meki/2 inhibition (Meki) has a natural phenotype, self-renewal ability, and enhanced pluripotency and proliferation. It is also achieved by regulating metabolism without affecting T cell receptor-mediated activation. DNA methylation analysis showed that Meki-induced T_SCM_ cells exhibited plasticity and loci-specific profiles, similar to those of T_SCM_ truly isolated from healthy donors, and had similar characteristics to naive and T_CM_ cells. Meki treatment of tumor-bearing mice also showed strong immune-mediated antitumor effects (86). These studies indicate that the regulation of glycolysis and metabolism is the key factor in inducing the formation of T_SCM_. Therefore, targeted metabolic checkpoints can make T cells differentiate into memory and provide more young T cells for immunotherapy (74, 81, 82, 86).

### The Molecules of Exhausted T Cells

T cell exhaustion is a phenomenon widely observed in humans. T_SCM_ or CAR-modified T_SCM_ expresses high levels of PD-1, TIM-3 or CTLA-4 after infiltrating the tumor, indicating that they have become exhausted T cells. Mostly due to T cell exhaustion and dysfunction by continuous TCR and cytokine stimulation. In addition, the effect of immune checkpoint inhibitors is very dependent on endogenous T cell function. However, they cannot reverse the exhaustion of T cells in cells that have undergone epigenetic changes. Therefore, this limits the long-term efficacy and wide application of cancer immunotherapy. Therefore, an in-depth understanding of the mechanism of T cell exhaustion is necessary for the study of T_SCM_ and its better clinical application. The term “ exhausted T cells” was originally derived from a mouse model of LCMV. It is now widely used to define the dysfunction state of T cells under chronic infection or tumor-induced long-term high antigen load stimulation (87). Enhanced and sustained T cell receptor stimulation is a key driver of T cell exhaustion. In recent years, the definition and identification of exhausted T cells have been divided from phenotype to transcriptional and epigenetic levels (88–90). Exhausted T cells are characterized by increased expression levels of inhibitory receptors such as PD-1, LAG3, 2B4, TIM-3 and CD28, and the gradual loss of effector functions, including impaired ability to secrete IFN-γ and tumor necrosis factor (91–95). PD-1 is mainly expressed on the surface of activated T cells and can inhibit T cell activation and proliferation. It is an important immunosuppressive molecule that plays an important role in suppressing immune responses and promoting self-tolerance (96–98). Programmed cell death ligand 1 (PD-L1) is a transmembrane protein, which is mainly expressed on the surface of antigen-presenting cells (APCs) such as dendritic cells (DCs), and can also be expressed on the surface of cancer cells and tumor infiltrating lymphocytes (TIL) (99–102). TOX is a nuclear DNA binding protein. TOX plays an important role in the development of thymus CD4^+^ T cells, NK cells and intrinsic lymphocytes, and is critical in the differentiation of tumor-specific T cells. Recent studies have described the important role of TOX in the differentiation of exhaustive CD8^+^ T cells and its molecular mechanism. It is unanimously found that the high expression of TOX is related to the high expression of a variety of inhibitory receptors (PD-1, TIM-3, TIGIT, CTLA-4, etc.) and the low expression of TCF1 (103). So inhibiting TOX expression may hinder the exhaustion of T cells (104–109). Many laboratories have identified a kind of exhausted T cell precursors (TPEX), which highly express molecules related to memory T cells, such as TCF1. TCF1 is a transcription factor and histone deacetylase (HDAC), which is related to the formation of T cell memory. Through single-cell RNA sequencing (scRNA-seq) and lineage tracing, the TCF1^+^Ly108^+^PD-1^+^CD8^+^ T cell population was identified. It was found that PD-1 stabilized the TCF1^+^TeX precursor cell pool and confirmed that PD-1 was this early stage protector of the TCF1 population (91, 110). Exhaustion first appeared in TCF1^+^ precursor T cells and then spread to the antigen-specific T cell pool. These findings will be important in the future to further investigate the developmental relationships in the later stages of exhaustion (111, 112).

**Figure 4:**
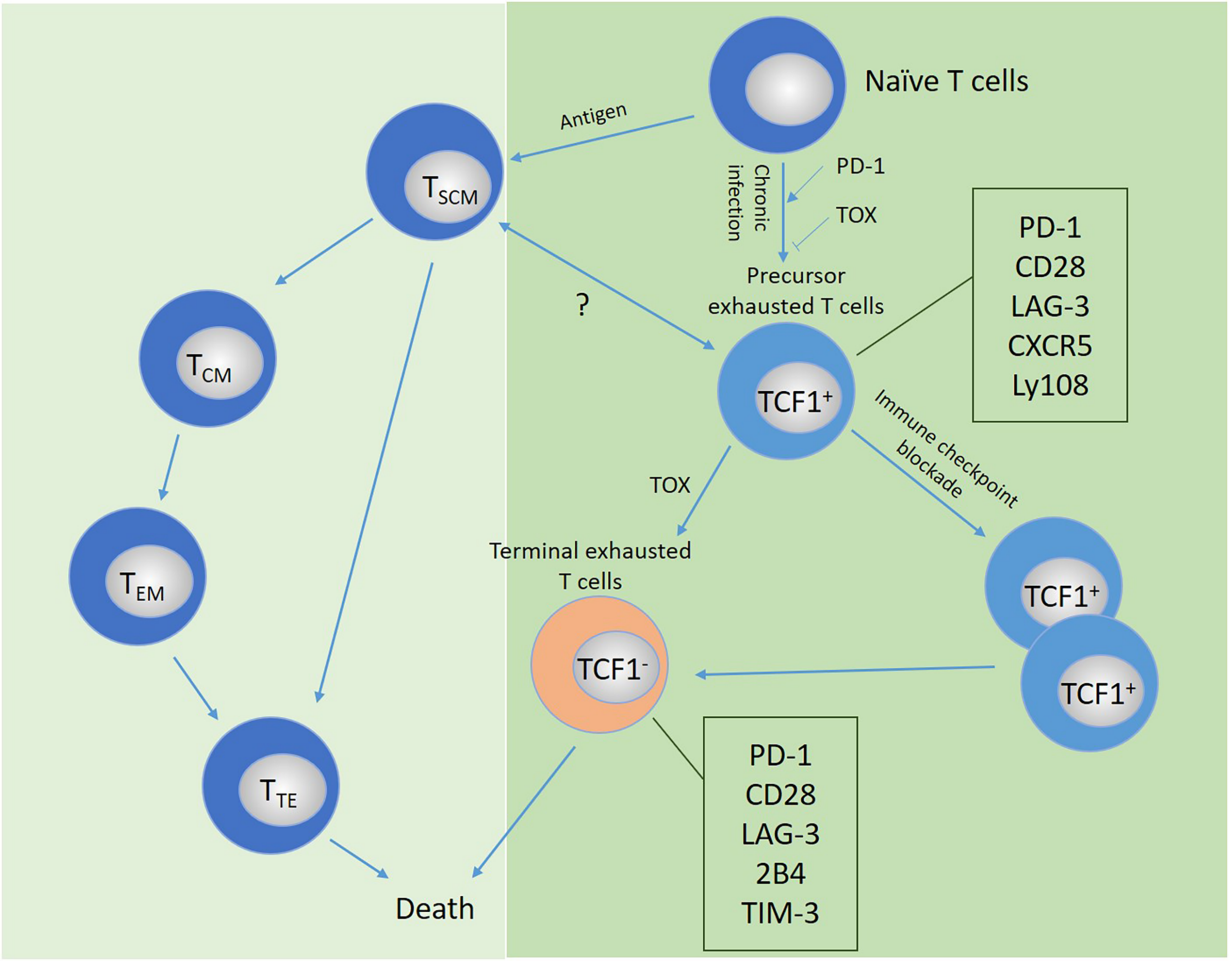
Possible developmental trajectory of exhausted T cells and the comparison and relationship with memory or effector T cells. Under continuous antigen stimulation, T cells transform from precursor exhausted cells into terminally exhausted T cell populations, which mainly depends on the expression of the transcription factor TCF-1, accompanied by the high expression of a variety of inhibitory receptors. The relationship between the differentiation of T cell subsets and exhausted T cells remains to be explored. PD-1, PD ligand 1; TCF1, T cell factor-1; TIM-3, T-cell immunoglobulin domain and mucin domain protein 3; LAG-3, lymphocyte activation gene 3; TOX, thymocyte selection-associated high-motility group (HMG) box protein.

At present, the specific mechanism of T cell exhaustion has not been fully elucidated. T cell exhaustion may be a parallel process with T cell differentiation. T cells at any stage of differentiation can be induced into exhausted T cells, which involves changes in different phenotypes and molecules. Excessive stimulation of precursor cells may be the origin of T cell failure. Under chronic infection or long-term tumor antigen stimulation, memory T cells and exhausted T cell precursors show different differentiation characteristics. Whether there is a link between the differentiation between these two subgroups should be a priority research area in the future. The possible potential developmental trajectories of exhausted T cells are shown in **Figure 4**.

At present, drugs for T cell exhaustion are still in the laboratory research or clinical trial stage. By reducing T cell exhaustion to promote the self-renewal ability and polyfunctionality of T_SCM_ cells (**Table 2**). Therefore, we do not know how to regulate the exhaustion process of T cells and reverse the exhausted state. Is it feasible to reach a certain effector state, and will there be side effects? Whether T_SCM_ can be designed to be exhaustion resistant? In general, the molecular mechanism of T_SCM_ cell formation is very complicated, and we describe them as clearly as possible in the review. More and more evidence supports the therapeutic potential of targeting exhausted T cells (115–118). We have already begun to understand the molecular mechanism of T cell exhaustion and early memory formation. Transforming exhausted T cells into rejuvenated T_SCM_ cells is the goal of our research.

**Table 2:** Key discoveries in the formation of induced culture T_SCM_.

Year	Authors	Discovery
**2013**	Nicoletta Cieri et al.	IL-7 and IL-15 instructed the generation of human memory stem T cells from naïve precursors (29).
**2016**	Lenka V. Hurton et al.	IL-15 could maintain the long-term persistence of CAR-T modified T_SCM_ (48, 57).
**2016**	Godehard Scholz et al.	Promote the generation of T_SCM_ by inhibiting the mTORC1 pathway (39, 84).
**2016**	Alvarez-Fernandez, C et al.	IL-21, IL-7 and IL-15 could effectively promote the generation of T_SCM_ under short anti-CD3/CD28 costimulation (113).
**2017**	T aisuke Kondo et al.	Coculture of activated T cells and stromal cells expressing Notch ligand could produce T_SCM_ cells with low expression of inhibitory receptors (89).
**2017**	TANJA KAARTINEN et al.	Simply reducing the amount of IL-2 could promote the generation of T_SCM_ (51).
**2018**	Charlotte M. Mousset et al.	AKT inhibitors promoted the *in vitro* generation of T_SCM_-like CD8^+^ T cells with a unique metabolic profile and retained polyfunctionality (79).
**2018**	Taisuke Kondo et al.	The coculture of activated T cells with IL-7, IL-15 and op9-hdll1 cells could effectively generate T_SCM_ cells (58).
**2018**	Yingshi Chen et al.	IL-21 promoted the generation of T_SCM_ cells more effectively than other common γ-chain cytokines (64).
**2020**	Taisuke Kondo et al.	The Notch-foxm1 axis played a key role in the metabolism of CAR-T modified T_SCM_ (74).
**2020**	Dalton Hermans et al.	LDH inhibition combined with IL-21 increase the formation of T_SCM_ cells (66).
**2020**	Pilipow, K et al.	Promote the formation of T_SCM_ by adding antioxidants (114).
**2021**	Ying Li et al.	IL-21 fusion anti-PD-1 antibody promoted the generation of T_SCM_ (67).
**2021**	Vivek Verma et al.	Meki was confirmed to induce reprogramming of CD8^+^ T cells into T_SCM_ (86).

Op9-hdll1, op9 cells expressing notch ligand, delta-like 1; Foxm1, forkhead box m1.

## Clinical Application

### The Antitumor Effect of T_SCM_

T_SCM_ cells are the least differentiated cells located at the top of the memory T lymphocyte hierarchy system. Compared with other T cells, they have stronger self-renewal ability and anti-tumor ability (84, 119, 120). As early as in previous studies, it has been found that T_SCM_ is considered a key determinant of immune memory and is involved in diversification of immune memory after allogeneic HSCT (32, 33). Play an important role in adult T-cell leukemia (34). With the FDA approval of CAR-T cell therapy for hematological malignancies, ACT has become a hot spot of continuous attention (63, 121–128). The clinical application of T_SCM_ cells is hindered because they are relatively rare in the circulation. According to reports, the CAR-T cell-modified T_SCM_ was cocultured with IL-2, IL-7 or IL-15 and then injected intravenously into tumor-bearing mice. It was found that the CAR-T/IL-15 group have the best anti-tumor effect (57). Guan et al. (129) prepared allogeneic antigen-specific CD8^+^ T_SCM_. It showed a proliferation history and rapidly differentiated into effector cells upon the E007 [the EB virus (EBV) transformed B lymphoblastoid cell lines (LCLs)] re-stimulation. Importantly, the prepared T_SCM_ cells could survive for a long time and reconstituted other T cell subsets *in vivo*, and could effectively eliminate E007 cells after being transferred to LCL burden mice. KUN et al. (120) presented a novel tumor therapeutic modality of the cryo-thermal therapy. After 90 days of cryo-thermal therapy, it can enhance the cytolytic function of CD8^+^ T cells, induce CD8^+^ T cells to differentiate into T_SCM_, and CD4^+^ T cells to differentiate into dominant CD4^-^ CTL, Th1 and TFH subets. Cryo-thermal therapy not only inhibits lung metastasis, but also promotes the regression of implanted melanoma and prolongs survival time (35, 130, 131). It was found that after antigen chimeric modification of T_SCM_, CD19-specific CAR T cell adoptive transfer has a significant antitumor effect on leukemia and lymphoma, and the therapeutic potential seems to be related to persistence *in vivo* (128, 129, 132, 133).

In SIP (an ex-vivo culture system modeled after the temporal changes of essential cytokines in an acute infection), TIL in the bone marrow of patients diagnosed with acute myeloid leukemia (AML) was treated with similar SIP, and it was found that these lymphocytes can be re-transformed into mutant CD45RA^+^ central memory T lymphocytes (T_CMRA_) with similar characteristics of T_SCM_. The expression of pro-inflammatory cytokines, TNF-α, IFN-γ and IL-2 increased, and T_CMRA_ also exhibited cytotoxicity against autologous AML blast cells (134). In addition, similar effects have been shown in the treatment of Hodgkin’s lymphoma. It showed a survival advantage, had higher tumor invasion and enhanced antitumor effect (133). Tumor immunotherapy is a promising treatment method. Transfect antigen-specific TCR gene or CAR vector to T_SCM_ to obtain CAR-T cells with poor differentiation and greater proliferation ability (135–139). A clinical trial study found that the genetically modified T_SCM_ can survive in the body for up to 12 years and has good safety and function (140). Recent studies have found that through integration site analysis, it is possible to study the fate of different types of CAR-T cells in patients, and it has been observed that T_SCM_ plays a central role in the early anti-leukemia response and late immune surveillance (38). This shows that this small portion of T cells is critical to the long-term success of CAR-T cell therapy. This new insight may help us improve CAR-T cell therapy and find out which patients are at higher risk of recurrence, and may benefit from stem cell transplantation after CAR-T cell therapy.

To date, CAR-T cells have achieved remarkable results in the treatment of hematological malignancies. However, despite extensive research, CAR-T cells have not been so successful in the treatment of solid tumors (141). Therefore, how to increase the trafficking and extravasation of T cells to the tumor sites and encourage the proliferation of T cells in the tumor is a problem that needs to be solved urgently. T_SCM_ have been shown to eradicate large tumors even when limited numbers of cells were transferred (28). Studies have found that chimeric T cells with multiple antigens may be a new direction for the treatment of solid tumors (71, 141, 142). At present, there are relatively few reports on the treatment of solid tumors with CAR-T-modified T_SCM_, so it is more challenging for CAR-T-modified T_SCM_ to target solid tumors. The future should be a priority research area. In summary, memory T cell subsets have good clinical application prospects in clinical antitumor immunotherapy, and can provide personalized treatment plans for improving the prognosis of patients (134, 143). In short, these studies provide a strong scientific basis and practical methods for the rapid advancement of T_SCM_ cells in clinical trials of human adoptive immunotherapy.

### The Importance of T_SCM_ in HIV-1 Immunotherapy and Vaccine Research

T_SCM_ cells play a key role in the pathogenesis of human immunodeficiency virus (HIV) infection (30, 144–146). The exhaustion of these cells will lead to the deterioration of the immune system and the development of AIDS. HIV-1 is an important part of the virus reservoirs. During HIV-1 infection, CD4^+^ T_SCM_ cells are confirmed to be the longest-lived HIV-1. Virus storage is one of the factors that cause persistent HIV-1 infection (147, 148). Therefore, CD4^+^ T_SCM_ cells can be used as a new target to clear the HIV-1 virus reservoir. The virus-latent cells are mainly concentrated in CD4^+^ T_SCM_. CD4^+^ T_SCM_ expresses lower levels of CCR5, but can still support the production and latent infection of R5-tropic HIV-1 (149, 150). In addition, CD4^+^ T_SCM_ is highly permissible for VSV-G-HIV-1 virus infection *in vitro*, and expresses relatively low levels of intracellular viral restriction factors, such as SAMHD1, Trim5alpha, and APOBEC3G. Moreover, these restriction factors can prevent HIV-1 from replicating in myeloid and dendritic cells (151–153). It was found that the CD4^+^ T_SCM_ of untreated HIV-1 infected persons contained high levels of HIV-1 RNA, which all indicated the sensitivity of CD4^+^ T_SCM_ cells to HIV-1. The study also found that in patients undergoing antiretroviral therapy (ART), CD4^+^ T_SCM_ cells also have viral DNA that can be activated. Moreover, among the subsets of CD4^+^ T memory cells, the number of HIV-1 DNA in T_SCM_ cells is the highest. During HIV infection, T cells play an important role in controlling virus replication. In patients receiving inhibitory antiretroviral therapy, CD8^+^ T_SCM_ with stem cell characteristics was found to be more abundant than untreated patients (154). In addition, prolonging the treatment time can increase the ratio of CD8^+^ T_SCM_, and preferentially secrete IL-2 under viral stimulation, indicating that CD8^+^ T_SCM_ is an important part of the cellular immune response to HIV-1. Able to maintain long-term, non-antigen-dependent cellular immune memory for HIV-1, which plays a key role in HIV control, but it seems unable to survive and proliferate during untreated infections (149). It is worth noting that HIV-1 specific CD8^+^ T_SCM_ cells may not directly participate in the antiviral process, but play a role by secreting IL-2 to maintain their own proliferation and differentiation (155–157). Recent studies have found that vaccination of the subtype C prophylactic HIV-1 vaccine candidate can induce more T_SCM_ and antiviral. Compared with MVA alone and placebo, it induces more peripheral CD8^+^ T_SCM_ cells and a higher level of CD8^+^ T cell-mediated inhibition of the replication of different HIV-1 branches can respond to acute HIV infection or effectively control the chronic replication of HIV (152). Recently, a cross-sectional study of 20 cases of HIV-infected patients on treatment alone and 20 cases of ART has revealed a new subset of CD4^+^ T cells: follicular regulatory T cells (TFR). The TFR of HIV^+^ patients had anti-apoptotic properties, high proliferation rate and T_SCM_-like properties, which leaded to the expansion of TFR, which in turn leaded to the dysfunction of TFH. Therefore, TFR cells may also become a new and potential therapeutic target for the treatment of HIV infection (158). How to target T_SCM_ therapy to provide new ideas for the development of new strategies for HIV-1 vaccines and immunotherapy still needs to continue to be explored and studied.

### T_SCM_ and Autoimmune Diseases

T_SCM_ cells provide long-term protective immunity for anti-tumor immunity, which is probably based on reactivity to self-antigens. Therefore, as a by-product of antitumor, T_SCM_-mediated autoimmunity is inevitable (18, 159). Recent related studies have reported that T_SCM_ cells are associated with a variety of autoimmune diseases. Systemic lupus erythematosus (SLE) is a chronic connective tissue disease involving multiple organs that occurs in young women. Compared with healthy controls, the percentage of CD4^+^ and CD8^+^ T_SCM_ cells in SLE patients increased significantly. Differentiated TFH cells increase the antibodies produced by their own B cells. T_SCM_ cells play a role in the pathogenesis of SLE by maintaining TFH cells (132). Moreover, compared with healthy controls, the CD4^+^ T_SCM_ of rheumatoid arthritis (RA) patients increased significantly (160). In the presence of IL-6, TCRs are easily activated to produce inflammatory cytokines. T_SCM_ cells may be a continuous source of the pathogenicity of RA (161). In patients with immune thrombocytopenia (ITP), the ratio of CD4^+^ and CD8^+^ T cells in the peripheral blood is unbalanced. The percentage of CD8^+^ T_SCM_ in peripheral blood of ITP patients was significantly reduced after glucocorticoid treatment, indicating that the imbalance of the ratio of CD8^+^ T_SCM_ may be involved in the occurrence and development of ITP (162). In addition, the frequency of acquired aplastic anemia (AA) CD8^+^ T_SCM_ after immunosuppressive treatment was significantly higher than that of healthy controls. The frequency of CD8^+^ T_SCM_ is also elevated in patients with autoimmune uveitis or sickle cell disease (130). B-cell-specific CD8^+^ T_SCM_ cells with high expression of glucose transporter 1 (GLUT1) can be detected in T1D patients. WZB117, a selective inhibitor of Gult-1, effectively inhibits T_SCM_ cells in type 1 diabetes (T1D) patients by inhibiting glucose metabolism (53). Long-term autoreactive or abnormally activated T_SCM_ cells may induce self-renewing inflammatory cell responses. Studies have found that rapamycin (mTORC1 inhibitor) is outstanding in the treatment of autoimmune diseases (163). The above studies indicate that T_SCM_ may be a potential therapeutic target for these autoimmune diseases. The possible role of T_SCM_ cells in other diseases with severe cellular immune response, such as autoimmune hepatitis, thyroiditis, and certain types of glomerulonephritis, is currently unclear, but represents a priority research area in the future.

## Conclusion

**Figure 5:**
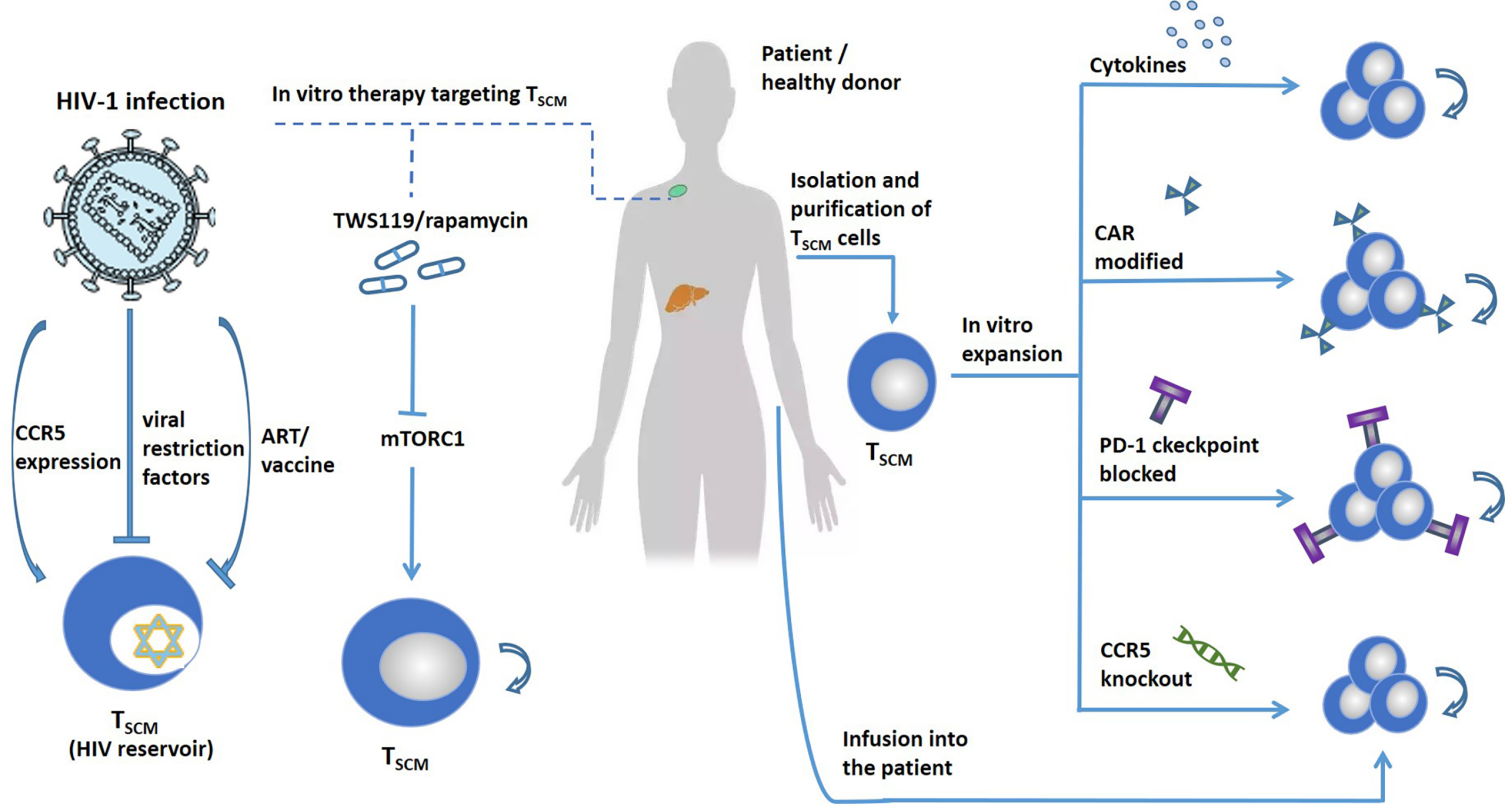
Target T_SCM_ cells to treat human diseases. T_SCM_ cells can exacerbate human disease. Left, treat T_SCM_-driven diseases, such as autoimmune diseases, HIV, etc., by blocking the production of T_SCM_. The expression of CCR5 promotes the infection of T_SCM_ cells with HIV. Viral restriction factors and vaccines can target T_SCM_ cells to treat HIV. Blocking the mTORC1 pathway promotes the self-renewal and differentiation of T_SCM_. Right, T_SCM_ cells are expanded *in vitro* by adding cytokines, CAR modification, immune checkpoint blocking, and gene editing. Stars represent cells latently infected with HIV.

T_SCM_ is a long-lived memory cell with self-renewal ability and multi-differentiation potential. Different subsets of memory T cells can be identified based on their surface markers, gene expression profiles, and metabolic methods. At the same time, clinical-grade memory T cells can be obtained through *in vitro* induction and culture for cell transfer. The formation of memory T cells in the body has been confirmed in pre-clinical trials. The genetically modified T_SCM_ can survive in the body for up to 12 years and has good safety and function (140, 164). Convincing evidence in mice and humans shows that T_SCM_ cells are an important tool for adoptive immunity in tumor immunotherapy (143, 162). On the contrary, it is precisely because of their powerful immune reconstruction ability that they play a double-edged role in human diseases, and they are also potential therapeutic targets for autoimmune diseases and HIV (**Figure 5**). However, there are still many problems that need to be solved, elucidating the molecular mechanism of maintaining the phenotype of T_SCM_ cells and the influence of epigenetic modification, how to obtain a sufficient number of clinical grade T_SCM_ for induction culture. The infused T_SCM_ cells are easily affected by the immune microenvironment and are difficult to exert antitumor effects, and how the T_SCM_ cells target the tumor site to kill tumor cells is a problem worthy of attention at present. CAR-modified T_SCM_ cells, although there is good preclinical evidence that they have anti-tumor activity, when they are intravenously infused into solid tumor patients, they still lack persistence and efficacy (71, 133, 142). At the same time, it is worth noting that a single treatment method cannot effectively eliminate tumor cells. Immune cell therapy should be combined with PD-1 monoclonal antibody, CTLA-4 monoclonal antibody or radiotherapy, chemotherapy and other treatment methods, so that patients can get better efficacy (165). T_SCM_ has long existed in the HIV-1 virus reservoir, so future research is necessary to determine whether the low virus accumulation in T_SCM_ cells represents a significant feature of HIV-1 infection. More effort is needed to clarify the changes between the different states of T_SCM_ cells in health and disease. Although significant progress has been made in tumor therapy, there is still a gap in our understanding of the role of T_SCM_ cells in autoimmunity and viral infections. 

## Author Contributions

YL designed the study and wrote the manuscript. DW and XY collected the literature. All authors contributed to the article and approved the submitted version.

## Funding

This work was funded by the Guangxi Science and Technology Research Base and Talent-specific Project (AD18126021); National Science and Technology Major Project for New Drug Innovation (2018ZX09733001-004-002); National Natural Science Foundation of China (NSFC) project (No. 81872491); Key Laboratory of the Ministry of Education Project for Early Prevention and Treatment of Regional High-risk Tumors (GKE2018-03, GKE2019-09, GKE-ZZ202007).

## Conflict of Interest

The authors declare that the research was conducted in the absence of any commercial or financial relationships that could be construed as a potential conflict of interest.

## Publisher’s Note

All claims expressed in this article are solely those of the authors and do not necessarily represent those of their affiliated organizations, or those of the publisher, the editors and the reviewers. Any product that may be evaluated in this article, or claim that may be made by its manufacturer, is not guaranteed or endorsed by the publisher.

## References

[1] Miller JD van der Most RG Akondy RS Glidewell JT Albott S Masopust D. Human Effector and Memory CD8+ T Cell Responses to Smallpox and Yellow Fever Vaccines. Immunity (2008) 28(5):710–22. doi: 10.1016/j.immuni.2008.02.020 18468462

[2] Figueroa JA Reidy A Mirandola L Trotter K Suvorava N Figueroa A. Chimeric Antigen Receptor Engineering: A Right Step in the Evolution of Adoptive Cellular Immunotherapy. Int Rev Immunol (2015) 34(2):154–87. doi: 10.3109/08830185.2015.1018419 25901860

[3] Khouri R Silva-Santos G Dierckx T Menezes SM Decanine D Theys K. A Genetic IFN/STAT1/FAS Axis Determines CD4 T Stem Cell Memory Levels and Apoptosis in Healthy Controls and Adult T-Cell Leukemia Patients. Oncoimmunology (2018) 7(5):e1426423. doi: 10.1080/2162402X.2018.1426423 29721391 PMC5927537

[4] Li X Chen W. Mechanisms of Failure of Chimeric Antigen Receptor T-Cell Therapy. Curr Opin Hematol (2019) 26(6):427–33. doi: 10.1097/MOH.0000000000000548 PMC6791516 31577606

[5] Martinez M Moon EK. CAR T Cells for Solid Tumors: New Strategies for Finding, Infiltrating, and Surviving in the Tumor Microenvironment. Front Immunol (2019) 10:128. doi: 10.3389/fimmu.2019.00128 30804938 PMC6370640

[6] Zhao J Song Y Liu D. Clinical Trials of Dual-Target CAR T Cells, Donor-Derived CAR T Cells, and Universal CAR T Cells for Acute Lymphoid Leukemia. J Hematol Oncol (2019) 12(1):17. doi: 10.1186/s13045-019-0705-x 30764841 PMC6376657

[7] Xu L Yao D Tan J He Z Yu Z Chen J. Memory T Cells Skew Toward Terminal Differentiation in the CD8+ T Cell Population in Patients With Acute Myeloid Leukemia. J Hematol Oncol (2018) 11(1):93. doi: 10.1186/s13045-018-0636-y 29986734 PMC6038290

[8] Thommen DS Schumacher TN. T Cell Dysfunction in Cancer. Cancer Cell (2018) 33(4):547–62. doi: 10.1016/j.ccell.2018.03.012 PMC7116508 29634943

[9] Sheng SY Gu Y Chuan Gang L Tang YY Yong Zou J Qing Zhang Y. The Characteristics of Naive-Like T Cells in Tumor-Infiltrating Lymphocytes From Human Lung Cancer. J Immunother (2017) 40(1):1–10. doi: 10.1097/CJI.0000000000000147 27828929

[10] Zheng C Zheng L Yoo JK Guo H Zhang Y Guo X. Landscape of Infiltrating T Cells in Liver Cancer Revealed by Single-Cell Sequencing. Cell (2017) 169(7):1342–56.e1316. doi: 10.1016/j.cell.2017.05.035 28622514

[11] Arcangeli S Falcone L Camisa B De Girardi F Biondi M Giglio F. Next-Generation Manufacturing Protocols Enriching TSCM CAR T Cells Can Overcome Disease-Specific T Cell Defects in Cancer Patients. Front Immunol (2020) 11:1217. doi: 10.3389/fimmu.2020.01217 32636841 PMC7317024

[12] Krishna S Lowery FJ Copeland AR Bahadiroglu E Mukherjee R Jia L. Stem-Like CD8 T Cells Mediate Response of Adoptive Cell Immunotherapy Against Human Cancer. Science (2020) 370(6522):1328–34. doi: 10.1126/science.abb9847 PMC8883579 33303615

[13] Vahidi Y Bagheri M Ghaderi A Faghih Z. CD8-Positive Memory T Cells in Tumor-Draining Lymph Nodes of Patients With Breast Cancer. BMC Cancer (2020) 20(1):257. doi: 10.1186/s12885-020-6714-x 32228503 PMC7106627

[14] Wu TD Madireddi S de Almeida PE Banchereau R Chen YJ Chitre AS. Peripheral T Cell Expansion Predicts Tumour Infiltration and Clinical Response. Nature (2020) 579(7798):274–8. doi: 10.1038/s41586-020-2056-8 32103181

[15] Liu Q Sun Z Chen L. Memory T Cells: Strategies for Optimizing Tumor Immunotherapy. Protein Cell (2020) 11(8):549–64. doi: 10.1007/s13238-020-00707-9 PMC7381543 32221812

[16] Zhang Y Joe G Hexner E Zhu J Emerson SG. Host-Reactive CD8+ Memory Stem Cells in Graft-*Versus*-Host Disease. Nat Med (2005) 11(12):1299–305. doi: 10.1038/nm1326 16288282

[17] Qian C W Y Cai H Laroye C De Carvalho Bittencourt M Clement L. Adenovirus-Specific T-Cell Subsets in Human Peripheral Blood and After IFN-γ Immunomagnetic Selection. J Immunother (Qian 2016) 39(1):27–35. doi: 10.1097/CJI.0000000000000105 26641259

[18] Gao S Liang X Wang H Bao B Zhang K Zhu Y. Stem Cell-Like Memory T Cells: A Perspective From the Dark Side. Cell Immunol (2021) 361:104273. doi: 10.1016/j.cellimm.2020.104273 33422699

[19] Xu L Zhang Y Luo G Li Y. The Roles of Stem Cell Memory T Cells in Hematological Malignancies. J Hematol Oncol (2015) 8:113. doi: 10.1186/s13045-015-0214-5 26462561 PMC4605076

[20] Lugli E Gattinoni L Roberto A Mavilio D Price DA Restifo NP. Identification, Isolation and *In Vitro* Expansion of Human and Nonhuman Primate T Stem Cell Memory Cells. Nat Protoc (2013) 8(1):33–42. doi: 10.1038/nprot.2012.143 23222456 PMC6328292

[21] Ando M Ito M Srirat T Kondo T Yoshimura A. Memory T Cell, Exhaustion, and Tumor Immunity. Immunol Med (2020) 43(1):1–9. doi: 10.1080/25785826.2019.1698261 31822213

[22] Morrot A. Human Stem Memory T Cells (TSCM) as Critical Players in the Long-Term Persistence of Immune Responses. Ann Transl Med (2017) 5(5):120. doi: 10.21037/atm.2017.02.28 28361085 PMC5360610

[23] Guo X Zhang Y Zheng L Zheng C Song J Zhang Q. Global Characterization of T Cells in Non-Small-Cell Lung Cancer by Single-Cell Sequencing. Nat Med (2018) 24(7):978–85. doi: 10.1038/s41591-018-0045-3 29942094

[24] Chebloune Y Moussa M Arrode-Brusés G Ronfort C Bose D Gagnon J. A Single Lentivector DNA Based Immunization Contains a Late Heterologous SIVmac251 Mucosal Challenge Infection. Vaccine (2020) 38(21):3729–39. doi: 10.1016/j.vaccine.2020.03.053 32278522

[25] Fearon DT Manders P Wagner SD. Arrested Differentiation, The Self-Renewing Memory Lymphocyte, and Vaccination. Science (2001) 293(5528):248–50. doi: 10.1126/science.1062589 11452114

[26] Luckey CJ B D Goldrath AW Weissman IL Benoist C Mathis D. Memory T and Memory B Cells Share a Transcriptional Program of Self-Renewal With Long-Term Hematopoietic Stem Cells. Proc Natl Acad Sci USA (2006) 103(9):3304–9. doi: 10.1073/pnas.0511137103 PMC1413911 16492737

[27] Gattinoni L Zhong XS Palmer DC Ji Y Hinrichs CS Yu Z. Wnt Signaling Arrests Effector T Cell Differentiation and Generates CD8+ Memory Stem Cells. Nat Med (2009) 15(7):808–13. doi: 10.1038/nm.1982 PMC2707501 19525962

[28] Gattinoni L Lugli E Ji Y Pos Z Paulos CM Quigley MF. A Human Memory T Cell Subset With Stem Cell-Like Properties. Nat Med (2011) 17(10):1290–7. doi: 10.1038/nm.2446 PMC3192229 21926977

[29] Cieri N Camisa B Cocchiarella F Forcato M Oliveira G Provasi E. IL-7 and IL-15 Instruct the Generation of Human Memory Stem T Cells From Naive Precursors. Blood (2013) 121(4):573–84. doi: 10.1182/blood-2012-05-431718 23160470

[30] Buzon MJ Sun H Li C Shaw A Seiss K Ouyang Z. HIV-1 Persistence in CD4+ T Cells With Stem Cell-Like Properties. Nat Med (2014) 20(2):139–42. doi: 10.1038/nm.3445 PMC3959167 24412925

[31] Graef P Buchholz VR Stemberger C Flossdorf M Henkel L Schiemann M. Serial Transfer of Single-Cell-Derived Immunocompetence Reveals Stemness of CD8(+) Central Memory T Cells. Immunity (2014) 41(1):116–26. doi: 10.1016/j.immuni.2014.05.018 25035956

[32] Roberto A Castagna L Zanon V Bramanti S Crocchiolo R McLaren JE. Role of Naive-Derived T Memory Stem Cells in T-Cell Reconstitution Following Allogeneic Transplantation. Blood (2015) 125(18):2855–64. doi: 10.1182/blood-2014-11-608406 PMC4424633 25742699

[33] Cieri N Oliveira G Greco R Forcato M Taccioli C Cianciotti B. Generation of Human Memory Stem T Cells After Haploidentical T-Replete Hematopoietic Stem Cell Transplantation. Blood (2015) 125(18):2865–74. doi: 10.1182/blood-2014-11-608539 25736310

[34] Nagai Y Kawahara M Hishizawa M Shimazu Y Sugino N Fujii S. T Memory Stem Cells are the Hierarchical Apex of Adult T-Cell Leukemia. Blood (2015) 125(23):3527–35. doi: 10.1182/blood-2014-10-607465 25847015

[35] Sabatino M Hu J Sommariva M Gautam S Fellowes V Hocker JD. Generation of Clinical-Grade CD19-Specific CAR-Modified CD8+ Memory Stem Cells for the Treatment of Human B-Cell Malignancies. Blood (2016) 128(4):519–28. doi: 10.1182/blood-2015-11-683847 PMC4965906 27226436

[36] Noviello M Manfredi F Ruggiero E Perini T Oliveira G Cortesi F. Bone Marrow Central Memory and Memory Stem T-Cell Exhaustion in AML Patients Relapsing After HSCT. Nat Commun (2019) 10(1):1065. doi: 10.1038/s41467-019-08871-1 30911002 PMC6434052

[37] Galletti G De Simone G Mazza EMC Puccio S Mezzanotte C Bi TM. Two Subsets of Stem-Like CD8(+) Memory T Cell Progenitors With Distinct Fate Commitments in Humans. Nat Immunol (2020) 21(12):1552–62. doi: 10.1038/s41590-020-0791-5 PMC7610790 33046887

[38] Biasco L Izotova N Rivat C Ghorashian S Richardson R Guvenel A. Clonal Expansion of T Memory Stem Cells Determines Early Anti-Leukemic Responses and Long-Term CAR T Cell Persistence in Patients. Nat Cancer (2021) 2(6):629–42. doi: 10.1038/s43018-021-00207-7 PMC7611448 34345830

[39] Yan C Chang J Song X Yan F Yu W An Y. Memory Stem T Cells Generated by Wnt Signaling From Blood of Human Renal Clear Cell Carcinoma Patients. Cancer Biol Med (2019) 16(1):109–24. doi: 10.20892/j.issn.2095-3941.2018.0118 PMC6528452 31119051

[40] Costa Del Amo P Lahoz-Beneytez J Boelen L Ahmed R Miners KL Zhang Y. Human TSCM Cell Dynamics *In Vivo* Are Compatible With Long-Lived Immunological Memory and Stemness. PloS Biol (2018) 16(6):e2005523. doi: 10.1371/journal.pbio.2005523 29933397 PMC6033534

[41] Good Z Borges L Vivanco Gonzalez N Sahaf B Samusik N Tibshirani R. Proliferation Tracing With Single-Cell Mass Cytometry Optimizes Generation of Stem Cell Memory-Like T Cells. Nat Biotechnol (2019) 37(3):259–66. doi: 10.1038/s41587-019-0033-2 PMC6521980 30742126

[42] Zhao Y Cai C Samir J Palgen JL Keoshkerian E Li H. Human CD8 T-Stem Cell Memory Subsets Phenotypic and Functional Characterization are Defined by Expression of CD122 or CXCR3. Eur J Immunol (2021) 51(7):1732–47. doi: 10.1002/eji.202049057 33844287

[43] Duckworth BC Lafouresse F Wimmer VC Broomfield BJ Dalit L Alexandre YO. Effector and Stem-Like Memory Cell Fates are Imprinted in Distinct Lymph Node Niches Directed by CXCR3 Ligands. Nat Immunol (2021) 22(4):434–48. doi: 10.1038/s41590-021-00878-5 33649580

[44] Santegoets SJAM Turksma AW Suhoski MM Stam AGM Albelda SM Hooijberg E. IL-21 Promotes the Expansion of CD27+ CD28+ Tumor Infiltrating Lymphocytes With High Cytotoxic Potential and Low Collateral Expansion of Regulatory T Cells. Transl Med (2013) 11(1):37. doi: 10.1186/1479-5876-11-37 PMC3626797 23402380

[45] Becker TC Wherry EJ Boone D Murali-Krishna K Antia R Ma A. Interleukin 15 Is Required for Proliferative Renewal of Virus-Specific Memory CD8 T Cells. J Exp Med (2002) 195(12):1541–8. doi: 10.1084/jem.20020369 PMC2193552 12070282

[46] Yi JS Du M Zajac AJ. A Vital Role for Interleukin-21 in the Control of a Chronic Viral Infection. Science (2009) 324(5934):1572–6. doi: 10.1126/science.1175194 PMC2736049 19443735

[47] Hashimoto M Im SJ Araki K Ahmed R. Cytokine-Mediated Regulation of CD8 T-Cell Responses During Acute and Chronic Viral Infection. Cold Spring Harb Perspect Biol (2019) 11(1):a028464. doi: 10.1101/cshperspect.a028464 29101105 PMC6314063

[48] Hurton LV Singh H Najjar AM Switzer KC Mi T Maiti S. Tethered IL-15 Augments Antitumor Activity and Promotes a Stem-Cell Memory Subset in Tumor-Specific T Cells. Proc Natl Acad Sci USA (2016) 113(48):E7788–97. doi: 10.1073/pnas.1610544113 PMC5137758 27849617

[49] Martkamchan S Onlamoon N Wang S Pattanapanyasat K Ammaranond P. The Effects of Anti-CD3/CD28 Coated Beads and IL-2 on Expanded T Cell for Immunotherapy. Adv Clin Exp Med (2016) 25(5):821–8. doi: 10.17219/acem/35771 28028943

[50] Perdomo-Celis F Medina-Moreno S Davis H Bryant J Taborda NA Rugeles MT. Characterization of CXCR5(+) CD8(+) T-Cells in Humanized NSG Mice. Immunobiology (2020) 225(2):151885. doi: 10.1016/j.imbio.2019.11.020 31836302

[51] Kaartinen T Luostarinen A Maliniemi P Keto J Arvas M Belt H. Low Interleukin-2 Concentration Favors Generation of Early Memory T Cells Over Effector Phenotypes During Chimeric Antigen Receptor T-Cell Expansion. Cytotherapy (2017) 19(6):689–702. doi: 10.1016/j.jcyt.2017.03.067 28411126

[52] Gattinoni L Klebanoff CA Palmer DC Wrzesinski C Kerstann K Yu Z. Acquisition of Full Effector Function *In Vitro* Paradoxically Impairs the *In Vivo* Antitumor Efficacy of Adoptively Transferred CD8+ T Cells. J Clin Invest (2005) 115(6):1616–26. doi: 10.1172/JCI24480 PMC1137001 15931392

[53] Vignali D Cantarelli E Bordignon C Canu A Citro A Annoni A. Detection and Characterization of CD8 + Autoreactive Memory Stem T Cells in Patients With Type 1 Diabetes. Diabetes (2018) 67(5):936–45. doi: 10.2337/db17-1390 29506985

[54] Abdelsamed HA Moustaki A Fan Y Dogra P Ghoneim HE Zebley CC. Human Memory CD8 T Cell Effector Potential Is Epigenetically Preserved During *In Vivo* Homeostasis. J Exp Med (2017) 214(6):1593–606. doi: 10.1084/jem.20161760 PMC5461005 28490440

[55] Adachi K Kano Y Nagai T Okuyama N Sakoda Y Tamada K. IL-7 and CCL19 Expression in CAR-T Cells Improves Immune Cell Infiltration and CAR-T Cell Survival in the Tumor. Nat Biotechnol (2018) 36(4):346–51. doi: 10.1038/nbt.4086 29505028

[56] Zanon V Lugli E. Differentiation of Diverse Progenies of Memory T Cells From Naive CD8(+) T Cell Precursors. Methods Mol Biol (2017) 1514:103–10. doi: 10.1007/978-1-4939-6548-9_8 27787795

[57] Alizadeh D Wong RA Yang X Wang D Pecoraro JR Kuo CF. IL15 Enhances CAR-T Cell Antitumor Activity by Reducing Mtorc1 Activity and Preserving Their Stem Cell Memory Phenotype. Cancer Immunol Res (2019) 7(5):759–72. doi: 10.1158/2326-6066.CIR-18-0466 PMC6687561 30890531

[58] Kondo T Imura Y Chikuma S Hibino S Omata-Mise S Ando M. Generation and Application of Human Induced-Stem Cell Memory T Cells for Adoptive Immunotherapy. Cancer Sci (2018) 109(7):2130–40. doi: 10.1111/cas.13648 PMC6029822 29790621

[59] Herda S Heimann A Obermayer B Ciraolo E Althoff S Russ J. Long-Term *In Vitro* Expansion Ensures Increased Yield of Central Memory T Cells as Perspective for Manufacturing Challenges. Int J Cancer (2021) 148(12):3097–110. doi: 10.1002/ijc.33523 33600609

[60] Manfredi F Abbati D Cianciotti BC Stasi L Potenza A Ruggiero E. Flow Cytometry Data Mining by Cytochain Identifies Determinants of Exhaustion and Stemness in TCR-Engineered T Cells. Eur J Immunol (2021) 51(8):1992–2005. doi: 10.1002/eji.202049103 34081326

[61] Castella M Caballero-Banos M Ortiz-Maldonado V Gonzalez-Navarro EA Sune G Antonana-Vidosola A. Point-Of-Care CAR T-Cell Production (ARI-0001) Using a Closed Semi-Automatic Bioreactor: Experience From an Academic Phase I Clinical Trial. Front Immunol (2020) 11:482. doi: 10.3389/fimmu.2020.00482 32528460 PMC7259426

[62] Fröhlich A Kisielow J Schmitz I Freigang S Shamshiev AT Weber J. IL-21R on T Cells Is Critical for Sustained Functionality and Control of Chronic Viral Infection. Science (2009) 324(5934):1576–80. doi: 10.1126/science.1172815 19478140

[63] Wu S Zhu W Peng Y Wang L Hong Y Huang L. The Antitumor Effects of Vaccine-Activated CD8(+) T Cells Associate With Weak TCR Signaling and Induction of Stem-Like Memory T Cells. Cancer Immunol Res (2017) 5(10):908–19. doi: 10.1158/2326-6066.CIR-17-0016 PMC5626646 28851693

[64] Chen Y Y F Jiang Y Chen J Wu K Chen X. Adoptive Transfer of Interleukin-21-Stimulated Human CD8+ T Memory Stem Cells Efficiently Inhibits Tumor Growth. Immunother (2018) 41(6):274–83. doi: 10.1097/CJI.0000000000000229 PMC6012057 29864078

[65] Cui W Liu Y Weinstein JS Craft J Kaech SM. An Interleukin-21-Interleukin-10-STAT3 Pathway Is Critical for Functional Maturation of Memory CD8+ T Cells. Immunity (2011) 35(5):792–805. doi: 10.1016/j.immuni.2011.09.017 22118527 PMC3431922

[66] Hermans D Gautam S Garcia-Canaveras JC Gromer D Mitra S Spolski R. Lactate Dehydrogenase Inhibition Synergizes With IL-21 to Promote CD8(+) T Cell Stemness and Antitumor Immunity. Proc Natl Acad Sci USA (2020) 117(11):6047–55. doi: 10.1073/pnas.1920413117 PMC7084161 32123114

[67] Li Y Cong Y Jia M He Q Zhong H Zhao Y. Targeting IL-21 to Tumor-Reactive T Cells Enhances Memory T Cell Responses and Anti-PD-1 Antibody Therapy. Nat Commun (2021) 12(1):951. doi: 10.1038/s41467-021-21241-0 33574265 PMC7878483

[68] Pearce EL Walsh MC Cejas PJ Harms GM Shen H Wang LS. Enhancing CD8 T-Cell Memory by Modulating Fatty Acid Metabolism. Nature (2009) 460(7251):103–7. doi: 10.1038/nature08097 PMC2803086 19494812

[69] Johnson MO Wolf MM Madden MZ Andrejeva G Sugiura A Contreras DC. Distinct Regulation of Th17 and Th1 Cell Differentiation by Glutaminase-Dependent Metabolism. Cell (2018) 175(7):1780–95.e1719. doi: 10.1016/j.cell.2018.10.001 30392958 PMC6361668

[70] Zhang JY Zhao YL Lv YP Cheng P Chen W Duan M. Modulation of CD8(+) Memory Stem T Cell Activity and Glycogen Synthase Kinase 3beta Inhibition Enhances Anti-Tumoral Immunity in Gastric Cancer. Oncoimmunology (2018) 7(4):e1412900. doi: 10.1080/2162402X.2017.1412900 29632726 PMC5889281

[71] Klampatsa A Dimou V Albelda SM. Mesothelin-Targeted CAR-T Cell Therapy for Solid Tumors. Expert Opin Biol Ther (2021) 21(4):473–86. doi: 10.1080/14712598.2021.1843628 33176519

[72] Sinclair LV Rolf J Emslie E Shi Y-B Taylor PM Cantrell DA. Control of Amino-Acid Transport by Antigen Receptors Coordinates the Metabolic Reprogramming Essential for T Cell Differentiation. Nat Immunol (2013) 14(5):500–8. doi: 10.1038/ni.2556 PMC3672957 23525088

[73] O'Sullivan D van der Windt GJ Huang SC Curtis JD Chang CH Buck MD. Memory CD8(+) T Cells Use Cell-Intrinsic Lipolysis to Support the Metabolic Programming Necessary for Development. Immunity (2014) 41(1):75–88. doi: 10.1016/j.immuni.2014.06.005 25001241 PMC4120664

[74] Kondo T Ando M Nagai N Tomisato W Srirat T Liu B. The NOTCH-FOXM1 Axis Plays a Key Role in Mitochondrial Biogenesis in the Induction of Human Stem Cell Memory-Like CAR-T Cells. Cancer Res (2020) 80(3):471–83. doi: 10.1158/0008-5472.CAN-19-1196 31767627

[75] Cui G Staron MM Gray SM Ho PC Amezquita RA Wu J. IL-7-Induced Glycerol Transport and TAG Synthesis Promotes Memory CD8+ T Cell Longevity. Cell (2015) 161(4):750–61. doi: 10.1016/j.cell.2015.03.021 PMC4704440 25957683

[76] Chang CH Pearce EL. Emerging Concepts of T Cell Metabolism as a Target of Immunotherapy. Nat Immunol (2016) 17(4):364–8. doi: 10.1038/ni.3415 PMC4990080 27002844

[77] Hu Z Zou Q Su B. Regulation of T Cell Immunity by Cellular Metabolism. Front Med (2018) 12(4):463–72. doi: 10.1007/s11684-018-0668-2 30112717

[78] Ma R Ji T Zhang H Dong W Chen X Xu P. A Pck1-Directed Glycogen Metabolic Program Regulates Formation and Maintenance of Memory CD8(+) T Cells. Nat Cell Biol (2018) 20(1):21–7. doi: 10.1038/s41556-017-0002-2 29230018

[79] Mousset CM Hobo W Ji Y Fredrix H De Giorgi V Allison RD. Ex Vivo AKT-Inhibition Facilitates Generation of Polyfunctional Stem Cell Memory-Like CD8(+) T Cells for Adoptive Immunotherapy. Oncoimmunology (2018) 7(10):e1488565. doi: 10.1080/2162402X.2018.1488565 30288356 PMC6169586

[80] Giuliani C. The Flavonoid Quercetin Induces AP-1 Activation in FRTL-5 Thyroid Cells. Antioxidants (Basel) (2019) 8(5):112. doi: 10.3390/antiox8050112 PMC6562732 31035637

[81] Kared H Tan SW Lau MC Chevrier M Tan C How W. Immunological History Governs Human Stem Cell Memory CD4 Heterogeneity via the Wnt Signaling Pathway. Nat Commun (2020) 11(1):821. doi: 10.1038/s41467-020-14442-6 32041953 PMC7010798

[82] Kolan SS Li G Wik JA Malachin G Guo S Kolan P. Cellular Metabolism Dictates T Cell Effector Function in Health and Disease. Scand J Immunol (2020) 92(5):e12956. doi: 10.1111/sji.12956 32767795

[83] Ma EH Poffenberger MC Wong AH Jones RG. The Role of AMPK in T Cell Metabolism and Function. Curr Opin Immunol (2017) 46:45–52. doi: 10.1016/j.coi.2017.04.004 28460345

[84] Scholz G Jandus C Zhang L Grandclement C Lopez-Mejia IC Soneson C. Modulation of mTOR Signalling Triggers the Formation of Stem Cell-Like Memory T Cells. EBioMedicine (2016) 4:50–61. doi: 10.1016/j.ebiom.2016.01.019 26981571 PMC4776068

[85] Geiger R Rieckmann JC Wolf T Basso C Feng Y Fuhrer T. L-Arginine Modulates T Cell Metabolism and Enhances Survival and Anti-Tumor Activity. Cell (2016) 167(3):829–42.e813. doi: 10.1016/j.cell.2016.09.031 27745970 PMC5075284

[86] Verma V Jafarzadeh N Boi S Kundu S Jiang Z Fan Y. MEK Inhibition Reprograms CD8(+) T Lymphocytes Into Memory Stem Cells With Potent Antitumor Effects. Nat Immunol (2021) 22(1):53–66. doi: 10.1038/s41590-020-00818-9 33230330 PMC10081014

[87] Utzschneider DT Charmoy M Chennupati V Pousse L Ferreira DP Calderon-Copete S. T Cell Factor 1-Expressing Memory-Like CD8(+) T Cells Sustain the Immune Response to Chronic Viral Infections. Immunity (2016) 45(2):415–27. doi: 10.1016/j.immuni.2016.07.021 27533016

[88] Wu T Ji Y Moseman EA Xu HC Manglani M Kirby M. The TCF1-Bcl6 Axis Counteracts Type I Interferon to Repress Exhaustion and Maintain T Cell Stemness. Sci Immunol (2016) 1(6):eaai8593. doi: 10.1126/sciimmunol.aai8593 28018990 PMC5179228

[89] Kondo T Morita R Okuzono Y Nakatsukasa H Sekiya T Chikuma S. Notch-Mediated Conversion of Activated T Cells Into Stem Cell Memory-Like T Cells for Adoptive Immunotherapy. Nat Commun (2017) 8:15338. doi: 10.1038/ncomms15338 28530241 PMC5458121

[90] Huang X Yang Y. Driving an Improved CAR for Cancer Immunotherapy. J Clin Invest (2016) 126(8):2795–8. doi: 10.1172/JCI88959 PMC4966332 27454296

[91] Chen Z Ji Z Ngiow SF Manne S Cai Z Huang AC. TCF-1-Centered Transcriptional Network Drives an Effector *Versus* Exhausted CD8 T Cell-Fate Decision. Immunity (2019) 51(5):840–55.e845. doi: 10.1016/j.immuni.2019.09.013 31606264 PMC6943829

[92] Raghu D Xue HH Mielke LA. Control of Lymphocyte Fate, Infection, and Tumor Immunity by TCF-1. Trends Immunol (2019) 40(12):1149–62. doi: 10.1016/j.it.2019.10.006 31734149

[93] He QF Xu Y Li J Huang ZM Li XH Wang X. CD8+ T-Cell Exhaustion in Cancer: Mechanisms and New Area for Cancer Immunotherapy. Brief Funct Genomics (2019) 18(2):99–106. doi: 10.1093/bfgp/ely006 29554204

[94] Kurachi M. CD8(+) T Cell Exhaustion. Semin Immunopathol (2019) 41(3):327–37. doi: 10.1007/s00281-019-00744-5 30989321

[95] Sawant DV Yano H Chikina M Zhang Q Liao M Liu C. Adaptive Plasticity of IL-10(+) and IL-35(+) Treg Cells Cooperatively Promotes Tumor T Cell Exhaustion. Nat Immunol (2019) 20(6):724–35. doi: 10.1038/s41590-019-0346-9 PMC6531353 30936494

[96] Siddiqui I Schaeuble K Chennupati V Fuertes Marraco SA Calderon-Copete S Pais Ferreira D. Intratumoral Tcf1(+)PD-1(+)CD8(+) T Cells With Stem-Like Properties Promote Tumor Control in Response to Vaccination and Checkpoint Blockade Immunotherapy. Immunity (2019) 50(1):195–211.e110. doi: 10.1016/j.immuni.2018.12.021 30635237

[97] Dosset M Joseph EL Rivera Vargas T Apetoh L. Modulation of Determinant Factors to Improve Therapeutic Combinations With Immune Checkpoint Inhibitors. Cells (2020) 9(7):1727. doi: 10.3390/cells9071727 PMC7408477 32707692

[98] Utzschneider DT Gabriel SS Chisanga D Gloury R Gubser PM Vasanthakumar A. Early Precursor T Cells Establish and Propagate T Cell Exhaustion in Chronic Infection. Nat Immunol (2020) 21(10):1256–66. doi: 10.1038/s41590-020-0760-z 32839610

[99] Saeidi A Zandi K Cheok YY Saeidi H Wong WF Lee CYQ. T-Cell Exhaustion in Chronic Infections: Reversing the State of Exhaustion and Reinvigorating Optimal Protective Immune Responses. Front Immunol (2018) 9:2569. doi: 10.3389/fimmu.2018.02569 30473697 PMC6237934

[100] Woroniecka K Chongsathidkiet P Rhodin K Kemeny H Dechant C Farber SH. T-Cell Exhaustion Signatures Vary With Tumor Type and Are Severe in Glioblastoma. Clin Cancer Res (2018) 24(17):4175–86. doi: 10.1158/1078-0432.CCR-17-1846 PMC6081269 29437767

[101] Blank CU Haining WN Held W Hogan PG Kallies A Lugli E. Defining 'T Cell Exhaustion'. Nat Rev Immunol (2019) 19(11):665–74. doi: 10.1038/s41577-019-0221-9 PMC7286441 31570879

[102] Fribourg M Anderson L Fischman C Cantarelli C Perin L La Manna G. T-Cell Exhaustion Correlates With Improved Outcomes in Kidney Transplant Recipients. Kidney Int (2019) 96(2):436–49. doi: 10.1016/j.kint.2019.01.040 PMC6650324 31040060

[103] Kim K Park S Park SY Kim G Park SM Cho JW. Single-Cell Transcriptome Analysis Reveals TOX as a Promoting Factor for T Cell Exhaustion and a Predictor for Anti-PD-1 Responses in Human Cancer. Genome Med (2020) 12(1):22. doi: 10.1186/s13073-020-00722-9 32111241 PMC7048139

[104] Heim K Binder B Sagar Wieland D Hensel N Llewellyn-Lacey S. TOX Defines the Degree of CD8+ T Cell Dysfunction in Distinct Phases of Chronic HBV Infection. Gut (2020) 70(8):1550–60. doi: 10.1136/gutjnl-2020-322404 PMC8292571 33097558

[105] Seo H Chen J Gonzalez-Avalos E Samaniego-Castruita D Das A Wang YH. TOX and TOX2 Transcription Factors Cooperate With NR4A Transcription Factors to Impose CD8(+) T Cell Exhaustion. Proc Natl Acad Sci USA (2019) 116(25):12410–5. doi: 10.1073/pnas.1905675116 PMC6589758 31152140

[106] Wang X He Q Shen H Xia A Tian W Yu W. TOX Promotes the Exhaustion of Antitumor CD8(+) T Cells by Preventing PD1 Degradation in Hepatocellular Carcinoma. J Hepatol (2019) 71(4):731–41. doi: 10.1016/j.jhep.2019.05.015 31173813

[107] Yao C Sun HW Lacey NE Ji Y Moseman EA Shih HY. Single-Cell RNA-Seq Reveals TOX as a Key Regulator of CD8(+) T Cell Persistence in Chronic Infection. Nat Immunol (2019) 20(7):890–901. doi: 10.1038/s41590-019-0403-4 31209400 PMC6588409

[108] Khan O Giles JR McDonald S Manne S Ngiow SF Patel KP. TOX Transcriptionally and Epigenetically Programs CD8(+) T Cell Exhaustion. Nature (2019) 571(7764):211–8. doi: 10.1038/s41586-019-1325-x PMC6713202 31207603

[109] Xu-Monette ZY Zhou J Young KH. PD-1 Expression and Clinical PD-1 Blockade in B-Cell Lymphomas. Blood (2018) 131(1):68–83. doi: 10.1182/blood-2017-07-740993 29118007 PMC5755041

[110] Zeng Z Wei F Ren X. Exhausted T Cells and Epigenetic Status. Cancer Biol Med (2020) 17(4):923–36. doi: 10.20892/j.issn.2095-3941.2020.0338 PMC7721092 33299644

[111] Beltra JC Manne S Abdel-Hakeem MS Kurachi M Giles JR Chen Z. Developmental Relationships of Four Exhausted CD8(+) T Cell Subsets Reveals Underlying Transcriptional and Epigenetic Landscape Control Mechanisms. Immunity (2020) 52(5):825–41.e828. doi: 10.1016/j.immuni.2020.04.014 32396847 PMC8360766

[112] Cai MC Zhao X Cao M Ma P Chen M Wu J. T-Cell Exhaustion Interrelates With Immune Cytolytic Activity to Shape the Inflamed Tumor Microenvironment. J Pathol (2020) 251(2):147–59. doi: 10.1002/path.5435 32222046

[113] Alvarez-Fernandez C Escriba-Garcia L Vidal S Sierra J Briones J. A Short CD3/CD28 Costimulation Combined With IL-21 Enhance the Generation of Human Memory Stem T Cells for Adoptive Immunotherapy. J Transl Med (2016) 14(1):214. doi: 10.1186/s12967-016-0973-y 27435312 PMC4952071

[114] Pilipow K Scamardella E Lugli E. Generating Stem-Like Memory T Cells With Antioxidants for Adoptive Cell Transfer Immunotherapy of Cancer. Methods Enzymol (2020) 631:137–58. doi: 10.1016/bs.mie.2019.08.016 31948545

[115] Tuluc F Spitsin S Tustin NB Murray JB Tustin R 3rd Schankel LA. Decreased PD-1 Expression on CD8 Lymphocyte Subsets and Increase in CD8 Tscm Cells in Children With HIV Receiving Raltegravir. AIDS Res Hum Retroviruses (2017) 33(2):133–42. doi: 10.1089/AID.2016.0108 PMC5312622 27615375

[116] Kallies A Zehn D Utzschneider DT. Precursor Exhausted T Cells: Key to Successful Immunotherapy? Nat Rev Immunol (2020) 20(2):128–36. doi: 10.1038/s41577-019-0223-7 31591533

[117] Terranova-Barberio M Pawlowska N Dhawan M Moasser M Chien AJ Melisko ME. Exhausted T Cell Signature Predicts Immunotherapy Response in ER-Positive Breast Cancer. Nat Commun (2020) 11(1):3584. doi: 10.1038/s41467-020-17414-y 32681091 PMC7367885

[118] Verdon DJ Mulazzani M Jenkins MR. Cellular and Molecular Mechanisms of CD8(+) T Cell Differentiation, Dysfunction and Exhaustion. Int J Mol Sci (2020) 21(19):7357. doi: 10.3390/ijms21197357 PMC7582856 33027962

[119] Kagoya Y Nakatsugawa M Yamashita Y Ochi T Guo T Anczurowski M. BET Bromodomain Inhibition Enhances T Cell Persistence and Function in Adoptive Immunotherapy Models. J Clin Invest (2016) 126(9):3479–94. doi: 10.1172/JCI86437 PMC5004946 27548527

[120] He K Liu P Xu LX. The Cryo-Thermal Therapy Eradicated Melanoma in Mice by Eliciting CD4(+) T-Cell-Mediated Antitumor Memory Immune Response. Cell Death Dis (2017) 8(3):e2703. doi: 10.1038/cddis.2017.125 28333145 PMC5386530

[121] Anderson KG Stromnes IM Greenberg PD. Obstacles Posed by the Tumor Microenvironment to T Cell Activity: A Case for Synergistic Therapies. Cancer Cell (2017) 31(3):311–25. doi: 10.1016/j.ccell.2017.02.008 PMC5423788 28292435

[122] He W Zhang H Han F Chen X Lin R Wang W. CD155T/TIGIT Signaling Regulates CD8(+) T-Cell Metabolism and Promotes Tumor Progression in Human Gastric Cancer. Cancer Res (2017) 77(22):6375–88. doi: 10.1158/0008-5472.CAN-17-0381 28883004

[123] Hashimoto M Kamphorst AO Im SJ Kissick HT Pillai RN Ramalingam SS. CD8 T Cell Exhaustion in Chronic Infection and Cancer: Opportunities for Interventions. Annu Rev Med (2018) 69:301–18. doi: 10.1146/annurev-med-012017-043208 29414259

[124] Choi BD Maus MV June CH Sampson JH. Immunotherapy for Glioblastoma: Adoptive T-Cell Strategies. Clin Cancer Res (2019) 25(7):2042–8. doi: 10.1158/1078-0432.CCR-18-1625 PMC6445734 30446589

[125] Atrash S Bano K Harrison B Abdallah AO. CAR-T Treatment for Hematological Malignancies. J Investig Med (2020) 68(5):956–64. doi: 10.1136/jim-2020-001290 32200355

[126] Abramson JS. Anti-CD19 CAR T-Cell Therapy for B-Cell Non-Hodgkin Lymphoma. Transfus Med Rev (2020) 34(1):29–33. doi: 10.1016/j.tmrv.2019.08.003 31677848

[127] Kasakovski D Xu L Li Y. T Cell Senescence and CAR-T Cell Exhaustion in Hematological Malignancies. J Hematol Oncol (2018) 11(1):91. doi: 10.1186/s13045-018-0629-x 29973238 PMC6032767

[128] Blaeschke F Stenger D Kaeuferle T Willier S Lotfi R Kaiser AD. Induction of a Central Memory and Stem Cell Memory Phenotype in Functionally Active CD4(+) and CD8(+) CAR T Cells Produced in an Automated Good Manufacturing Practice System for the Treatment of CD19(+) Acute Lymphoblastic Leukemia. Cancer Immunol Immunother (2018) 67(7):1053–66. doi: 10.1007/s00262-018-2155-7 PMC11028239 29605883

[129] Guan L Li X Wei J Liang Z Yang J Weng X. Antigen-Specific CD8+ Memory Stem T Cells Generated From Human Peripheral Blood Effectively Eradicate Allogeneic Targets in Mice. Stem Cell Res Ther (2018) 9(1):337. doi: 10.1186/s13287-018-1080-1 30526661 PMC6286512

[130] Hosokawa K Muranski P Feng X Townsley DM Liu B Knickelbein J. Memory Stem T Cells in Autoimmune Disease: High Frequency of Circulating CD8+ Memory Stem Cells in Acquired Aplastic Anemia. J Immunol (2016) 196(4):1568–78. doi: 10.4049/jimmunol.1501739 PMC4744506 26764034

[131] Wang Y Whittall T Neil S Britton G Mistry M Rerks-Ngarm S. A Novel Mechanism Linking Memory Stem Cells With Innate Immunity in Protection Against HIV-1 Infection. Sci Rep (2017) 7(1):1057. doi: 10.1038/s41598-017-01188-3 28432326 PMC5430909

[132] Lee YJ Park JA Kwon H Choi YS Jung KC Park SH. Role of Stem Cell-Like Memory T Cells in Systemic Lupus Erythematosus. Arthritis Rheumatol (2018) 70(9):1459–69. doi: 10.1002/art.40524 29660266

[133] Alvarez-Fernandez C Escriba-Garcia L Caballero AC Escudero-Lopez E Ujaldon-Miro C Montserrat-Torres R. Memory Stem T Cells Modified With a Redesigned CD30-Chimeric Antigen Receptor Show an Enhanced Antitumor Effect in Hodgkin Lymphoma. Clin Transl Immunol (2021) 10(4):e1268. doi: 10.1002/cti2.1268 PMC8082716 33968404

[134] Teo YWB Linn YC Goh YT Li S Ho LP. Tumor Infiltrating Lymphocytes From Acute Myeloid Leukemia Marrow can be Reverted to CD45RA+ Central Memory State by Reactivation in SIP (Simulated Infective Protocol). Immunobiology (2019) 224(4):526–31. doi: 10.1016/j.imbio.2019.05.001 31072628

[135] Hoffmann JM Schubert ML Wang L Huckelhoven A Sellner L Stock S. Differences in Expansion Potential of Naive Chimeric Antigen Receptor T Cells From Healthy Donors and Untreated Chronic Lymphocytic Leukemia Patients. Front Immunol (2017) 8:1956. doi: 10.3389/fimmu.2017.01956 29375575 PMC5767585

[136] Liu J Zhong JF Zhang X Zhang C. Allogeneic CD19-CAR-T Cell Infusion After Allogeneic Hematopoietic Stem Cell Transplantation in B Cell Malignancies. J Hematol Oncol (2017) 10(1):35. doi: 10.1186/s13045-017-0405-3 28143567 PMC5282795

[137] Mollanoori H Shahraki H Rahmati Y Teimourian S. CRISPR/Cas9 and CAR-T Cell, Collaboration of Two Revolutionary Technologies in Cancer Immunotherapy, an Instruction for Successful Cancer Treatment. Hum Immunol (2018) 79(12):876–82. doi: 10.1016/j.humimm.2018.09.007 30261221

[138] Agarwal S Weidner T Thalheimer FB Buchholz CJ. *In Vivo* Generated Human CAR T Cells Eradicate Tumor Cells. Oncoimmunology (2019) 8(12):e1671761. doi: 10.1080/2162402X.2019.1671761 31741773 PMC6844313

[139] Brudno JN Kochenderfer JN. Recent Advances in CAR T-Cell Toxicity: Mechanisms, Manifestations and Management. Blood Rev (2019) 34:45–55. doi: 10.1016/j.blre.2018.11.002 30528964 PMC6628697

[140] Biasco L Scala S Ricci LB Dionisio F Baricordi C Calabria A. *In Vivo* Tracking of T Cells in Humans Unveils Decade-Long Survival and Activity of Genetically Modified T Memory Stem Cells. Transl Med (2015) 7(273):273ra213. doi: 10.1126/sciimmunol.aay0555 25653219

[141] Jin L Tao H Karachi A Long Y Hou AY Na M. CXCR1- or CXCR2-Modified CAR T Cells Co-Opt IL-8 for Maximal Antitumor Efficacy in Solid Tumors. Nat Commun (2019) 10(1):4016. doi: 10.1038/s41467-019-11869-4 31488817 PMC6728370

[142] Choe JH W PB Simic MS Gilbert RD Li AW Krasnow NA. SynNotch-CAR T Cells Overcome Challenges of Specificity, Heterogeneity, and Persistence in Treating Glioblastoma. Sci Transl Med (2021) 13(591):eabe7378. doi: 10.1126/scitranslmed.abe7378 33910979 PMC8362330

[143] Padgett LE Dinh HQ Wu R Gaddis DE Araujo DJ Winkels H. Naive CD8(+) T Cells Expressing CD95 Increase Human Cardiovascular Disease Severity. Arterioscler Thromb Vasc Biol (2020) 40(12):2845–59. doi: 10.1161/ATVBAHA.120.315106 PMC8184121 33054398

[144] Flynn JK Gorry PR. Stem Memory T Cells (TSCM)-Their Role in Cancer and HIV Immunotherapies. Clin Transl Immunol (2014) 3(7):e20. doi: 10.1038/cti.2014.16 PMC4232066 25505968

[145] Flynn JK Paukovics G Cashin K Borm K Ellett A Roche M. Quantifying Susceptibility of CD4+ Stem Memory T-Cells to Infection by Laboratory Adapted and Clinical HIV-1 Strains. Viruses (2014) 6(2):709–26. doi: 10.3390/v6020709 PMC3939479 24517971

[146] Chahroudi A Silvestri G Lichterfeld M. T Memory Stem Cells and HIV: A Long-Term Relationship. Curr HIV/AIDS Rep (2015) 12(1):33–40. doi: 10.1007/s11904-014-0246-4 25578055 PMC4370789

[147] Calascibetta F Micci L Carnathan D Lawson B Vanderford TH Bosinger SE. Antiretroviral Therapy in Simian Immunodeficiency Virus-Infected Sooty Mangabeys: Implications for AIDS Pathogenesis. J Virol (2016) 90(16):7541–51. doi: 10.1128/JVI.00598-16 PMC4984638 27279614

[148] Cartwright EK Palesch D Mavigner M Paiardini M Chahroudi A Silvestri G. Initiation of Antiretroviral Therapy Restores CD4+ T Memory Stem Cell Homeostasis in Simian Immunodeficiency Virus-Infected Macaques. J Virol (2016) 90(15):6699–708. doi: 10.1128/JVI.00492-16 PMC4944279 27170752

[149] Vigano S Negron J Ouyang Z Rosenberg ES Walker BD Lichterfeld M. Prolonged Antiretroviral Therapy Preserves HIV-1-Specific CD8 T Cells With Stem Cell-Like Properties. J Virol (2015) 89(15):7829–40. doi: 10.1128/JVI.00789-15 PMC4505639 25995260

[150] Lu X Song B Weng W Xia H Su B Wu H. CD4(+) T Memory Stem Cells Correlate With Disease Progression in Chronically HIV-1-Infected Patients. Viral Immunol (2017) 30(9):642–8. doi: 10.1089/vim.2017.0017 29035156

[151] Guardo AC Zarama A Gonzalez T Bargallo ME Rojas J Martinez E. Effects on Immune System and Viral Reservoir of a Short-Cycle Antiretroviral Therapy in Virologically Suppressed HIV-Positive Patients. AIDS (2019) 33(6):965–72. doi: 10.1097/QAD.0000000000002169 30946150

[152] Munusamy Ponnan S Hayes P Fernandez N Thiruvengadam K Pattabiram S Nesakumar M. Evaluation of Antiviral T Cell Responses and TSCM Cells in Volunteers Enrolled in a Phase I HIV-1 Subtype C Prophylactic Vaccine Trial in India. PloS One (2020) 15(2):e0229461. doi: 10.1371/journal.pone.0229461 32097435 PMC7041807

[153] Manganaro L Hong P Hernandez MM Argyle D Mulder LCF Potla U. IL-15 Regulates Susceptibility of CD4(+) T Cells to HIV Infection. Proc Natl Acad Sci USA (2018) 115(41):E9659–67. doi: 10.1073/pnas.1806695115 PMC6187195 30257946

[154] Utay NS Vigil KJ Somasunderam A Aulicino PC Smulevitz B Chiadika S. Timing of Antiretroviral Therapy Initiation Determines Rectal Natural Killer Cell Populations. AIDS Res Hum Retroviruses (2020) 36(4):314–23. doi: 10.1089/AID.2019.0225 PMC7185341 31838858

[155] Finley J. Elimination of Cancer Stem Cells and Reactivation of Latent HIV-1 via AMPK Activation: Common Mechanism of Action Linking Inhibition of Tumorigenesis and the Potential Eradication of HIV-1. Med Hypotheses (2017) 104:133–46. doi: 10.1016/j.mehy.2017.05.032 28673572

[156] Fenwick C Joo V Jacquier P Noto A Banga R Perreau M. T-Cell Exhaustion in HIV Infection. Immunol Rev (2019) 292(1):149–63. doi: 10.1111/imr.12823 PMC7003858 31883174

[157] Sharov KS. HIV/SARS-CoV-2 Co-Infection: T Cell Profile, Cytokine Dynamics and Role of Exhausted Lymphocytes. Int J Infect Dis (2021) 102:163–9. doi: 10.1016/j.ijid.2020.10.049 PMC7585731 33115677

[158] Zhao S Xu W Tu B Hong WG Zhang Z Chen WW. Alterations of the Frequency and Functions of Follicular Regulatory T Cells and Related Mechanisms in HIV Infection. J Infect (2020) 81(5):776–84. doi: 10.1016/j.jinf.2020.09.014 32956725

[159] Roederer M Quaye L Mangino M Beddall MH Mahnke Y Chattopadhyay P. The Genetic Architecture of the Human Immune System: A Bioresource for Autoimmunity and Disease Pathogenesis. Cell (2015) 161(2):387–403. doi: 10.1016/j.cell.2015.02.046 25772697 PMC4393780

[160] Cianciotti BC Ruggiero E Campochiaro C Oliveira G Magnani ZI Baldini M. CD4+ Memory Stem T Cells Recognizing Citrullinated Epitopes Are Expanded in Patients With Rheumatoid Arthritis and Sensitive to Tumor Necrosis Factor Blockade. Arthritis Rheumatol (2020) 72(4):565–75. doi: 10.1002/art.41157 31682074

[161] Lee YJ Park EH Park JW Jung KC Lee EB. Proinflammatory Features of Stem Cell-Like Memory T Cells From Human Patients With Rheumatoid Arthritis. J Immunol (2021) 207(2):381–8. doi: 10.4049/jimmunol.2000814 34162725

[162] Cao J Zhang C Han X Cheng H Chen W Qi K. Emerging Role of Stem Cell Memory-Like T Cell in Immune Thrombocytopenia. Scand J Immunol (2019) 89(3):e12739. doi: 10.1111/sji.12739 30506564

[163] Bride KL Vincent T Smith-Whitley K Lambert MP Bleesing JJ Seif AE. Sirolimus Is Effective in Relapsed/Refractory Autoimmune Cytopenias: Results of a Prospective Multi-Institutional Trial. Blood (2016) 127(1):17–28. doi: 10.1182/blood-2015-07-657981 26504182 PMC4705607

[164] Flynn JK Gorry PR. T Cell Therapies-are T Memory Stem Cells the Answer? Ann Transl Med (2015) 3(17):251. doi: 10.3978/j.issn.2305-5839.2015.08.13 26605297 PMC4620096

[165] Morrot A. Lifelong Protection Mediated by Stem Cell-Like CD8(+) T Memory Subset Cells (Tscm) Induced by Vaccination. Ann Transl Med (2016) 4(11):221. doi: 10.21037/atm.2016.05.38 27386495 PMC4916359

